# Comparative Anatomy of Chromosomal Domains with Imprinted and Non-Imprinted Allele-Specific DNA Methylation

**DOI:** 10.1371/journal.pgen.1003622

**Published:** 2013-08-29

**Authors:** Anupam Paliwal, Alexis M. Temkin, Kristi Kerkel, Alexander Yale, Iveta Yotova, Natalia Drost, Simon Lax, Chia-Ling Nhan-Chang, Charles Powell, Alain Borczuk, Abraham Aviv, Ronald Wapner, Xiaowei Chen, Peter L. Nagy, Nicholas Schork, Catherine Do, Ali Torkamani, Benjamin Tycko

**Affiliations:** 1 Institute for Cancer Genetics, Columbia University Medical Center, New York, New York, United States of America; 2 Department of Obstetrics and Gynecology and Division of Fetal-Maternal Medicine, Columbia University Medical Center, New York, New York, United States of America; 3 Department of Medicine, Pulmonary Division, Columbia University Medical Center, New York, New York, United States of America; 4 Department of Pathology, Columbia University Medical Center, New York, New York, United States of America; 5 Center for Human Development and Aging, University of Medicine and Dentistry, New Jersey Medical School, Newark, New Jersey, United States of America; 6 Medical Science Division, Fox Chase Cancer Center, Philadelphia, Pennsylvania, United States of America; 7 Taub Institute for Research on Alzheimer's disease and the Aging Brain, Columbia University Medical Center, New York, New York, United States of America; 8 Scripps Translational Science Institute, La Jolla, California, United States of America; Beckman Research Institute, United States of America

## Abstract

Allele-specific DNA methylation (ASM) is well studied in imprinted domains, but this type of epigenetic asymmetry is actually found more commonly at non-imprinted loci, where the ASM is dictated not by parent-of-origin but instead by the local haplotype. We identified loci with strong ASM in human tissues from methylation-sensitive SNP array data. Two index regions (bisulfite PCR amplicons), one between the *C3orf27* and *RPN1* genes in chromosome band 3q21 and the other near the *VTRNA2-1* vault RNA in band 5q31, proved to be new examples of imprinted DMRs (maternal alleles methylated) while a third, between *STEAP3* and *C2orf76* in chromosome band 2q14, showed non-imprinted haplotype-dependent ASM. Using long-read bisulfite sequencing (bis-seq) in 8 human tissues we found that in all 3 domains the ASM is restricted to single differentially methylated regions (DMRs), each less than 2kb. The ASM in the *C3orf27-RPN1* intergenic region was placenta-specific and associated with allele-specific expression of a long non-coding RNA. Strikingly, the discrete DMRs in all 3 regions overlap with binding sites for the insulator protein CTCF, which we found selectively bound to the unmethylated allele of the *STEAP3*-*C2orf76* DMR. Methylation mapping in two additional genes with non-imprinted haplotype-dependent ASM, *ELK3* and *CYP2A7*, showed that the *CYP2A7* DMR also overlaps a CTCF site. Thus, two features of imprinted domains, highly localized DMRs and allele-specific insulator occupancy by CTCF, can also be found in chromosomal domains with non-imprinted ASM. Arguing for biological importance, our analysis of published whole genome bis-seq data from hES cells revealed multiple genome-wide association study (GWAS) peaks near CTCF binding sites with ASM.

## Author Summary

Allele-specific DNA methylation (ASM) is a central mechanism of gene regulation in humans, which can influence inter-individual differences in physical and mental traits and disease susceptibility. ASM is mediated either by parental imprinting, in which the repressed copy (allele) of the gene is determined by which type of parent (mother or father) transmitted it or, for a larger number of genes, by the local DNA sequence, independent of which parent transmitted it. Chromosomal regions with imprinted ASM have been well studied, and certain mechanistic principles, including the role of discrete differentially methylated regions (DMRs) and involvement of the insulator protein CTCF, have emerged. However, the molecular mechanisms underlying non-imprinted sequence-dependent ASM are not yet understood. Here we describe our detailed mapping of ASM across 5 gene regions, including two novel examples of imprinted ASM and three gene regions with non-imprinted, sequence-dependent ASM. Our data uncover shared molecular features – small discrete DMRs, and the binding of CTCF to these DMRs, in examples of both types of ASM. Combining ASM mapping with genetic association data suggests that sequence-dependent ASM at CTCF binding sites influences diverse human traits.

## Introduction

Evidence from genome-wide association studies (GWAS) and cross-species comparisons suggests that many inter-individual phenotypic differences result from genetic variants in non-coding DNA sequences. Thus a major challenge in the post-genomic era is to define the mechanisms by which non-coding sequence polymorphisms and haplotypes result in differences in biological phenotypes. One hypothesis comes from recent work that has revealed strong *cis*-acting influences of simple nucleotide polymorphisms (SNPs), and the haplotypes in which these SNPs are embedded, on epigenetic marks, leading to allele-specific DNA methylation (ASM) and allele-specific chromatin structure [1]. Historically, ASM has been most intensively studied in the context of genomic imprinting - a parent-of-origin dependent phenomenon that affects about 80 human genes. Mechanistic principles that have emerged from studying imprinted domains include the presence of discrete small (one to several kb) DNA intervals, called differentially methylated regions (DMRs), which show strong parent-of-origin dependent asymmetry in CpG methylation between the two alleles and which control the allele-specific expression (ASE) of one or more nearby genes in *cis*, in some cases via methylation-sensitive binding of the insulator protein CTCF [2]–[6].

While genomic imprinting is a potent mode of gene regulation, it affects fewer loci than the more recently recognized phenomenon of non-imprinted haplotype-dependent ASM. Mapping haplotype-dependent ASM, and related phenomena such as ASE, will be useful for interpreting the biological meaning of statistical peaks from GWAS (and non-coding variants from post-GWAS exome sequencing studies), as *bona fide* regulatory sequence variants can reveal their presence by conferring a measurable epigenetic asymmetry between the two alleles. However as yet there have not been many insights to the molecular mechanisms underlying non-imprinted ASM. This situation raises an interesting question – could any principles from studies of genomic imprinting also be relevant for understanding haplotype-dependent, non-imprinted, ASM? To begin to address this issue, and to identify and characterize new examples of loci with imprinted and non-imprinted ASM, we have searched for “index regions” in the human genome showing strong and highly recurrent ASM and used these locations as starting points for intensive local mapping of DNA methylation patterns in multiple human tissues. Here we describe these data for new examples of loci with imprinted and non-imprinted ASM, which reveal epigenomic features that are shared between these two allele-specific phenomena.

## Results

### Index regions with ASM identified by MSNP

**Figure 1:**
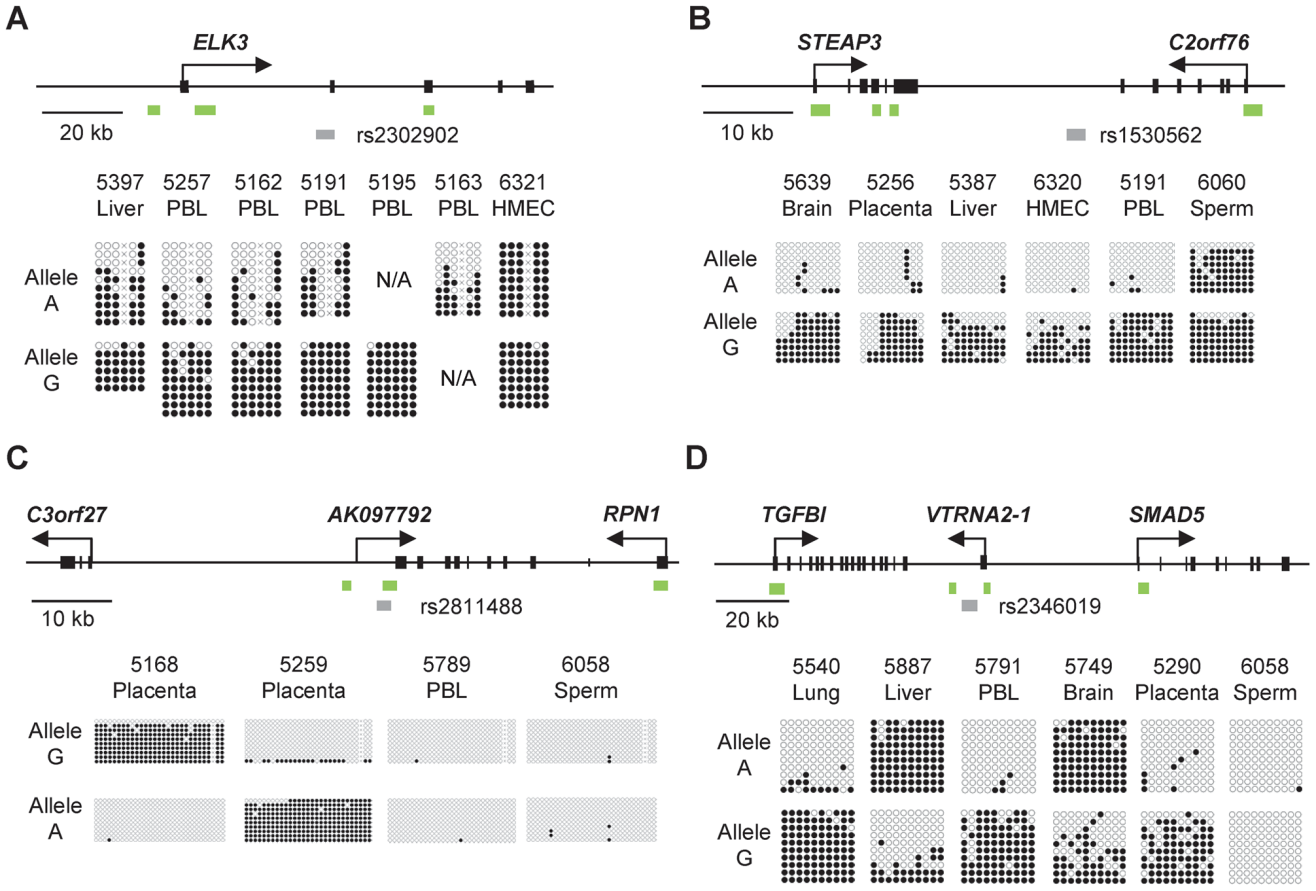
Bis-seq showing strong ASM in the *ELK3*, *STEAP3-C2orf76*, *C3orf27-RPN1*, and *VTRNA2-1* index regions. **A,** Gene map and bis-seq of the index amplicon containing SNP rs2302902 in the first intron of the *ELK3* gene. The strong asymmetry in CpG methylation, with the G allele consistently hypermethylated in the PBL samples, indicates haplotype-dependent non-imprinted ASM (additional data in Table 1; haplotype map in Figure S5). There is some tissue-specificity, with biallelic hypermethylation in the HMEC sample. The grey (lower) bars indicate the index amplicons for initial bis-seq, each tagged by the index SNP that showed recurrent ASM in the MSNP data. The green bars indicate CGIs and the black rectangles are exons. The X's indicate polymorphic CpG sites. **B,** Gene map and bis-seq of the index amplicon containing SNP rs1530562, between the *STEAP3* and *C2orf76* genes in chromosome band 2q14, showing strong ASM with the G allele consistently hypermethylated in multiple tissues consistent with haplotype-dependent non-imprinted ASM, but with biallelic hypermethylation in sperm DNA (additional data in Table 1). **C,** Gene map and bis-seq of the index amplicon containing SNP rs2811488 located downstream of the last exon of the *RPN1* gene in chromosome band 3q21, showing strong ASM in placenta, with the G allele or A allele hypermethylated, depending on parent-of-origin, as proven in Figure 2. For this imprinted DMR the ASM is highly tissue-specific, being seen in placenta but not in PBL, liver, lung, brain, HMEC or sperm (additional data in Table 1 and Figure S2). **D,** Gene map and bilsufite sequencing of the index amplicon containing SNP rs2346019 downstream of the *VTRNA2-1* vault-family RNA, located in chromosome band 5q31. Strong ASM is observed in multiple tissues with the A allele or G allele methylated, consistent with imprinting, which is proven by the data in Figure 2.

The MSNP procedure, an adaptation of SNP arrays for detecting ASM, was described in our initial report of haplotype-dependent ASM in human tissues [7]. As a starting point for comparing the structures of chromosomal domains with imprinted (parent-of-origin dependent) versus non-imprinted (haplotype dependent) ASM we applied higher resolution MSNP to several human tissue types from multiple individuals and identified 4 additional SNP-tagged *Sty*I or *Nsp*I restriction fragments with CpG dinucleotides in *Hpa*II sites that showed strongly asymmetrical methylation between the two alleles in multiple heterozygous samples (examples in Figure S1). These index fragments are tagged by SNPs rs1530562 between the *STEAP3* and *C2orf76* genes (chromosome band 2q14), rs2346019 near the *VTRNA2-1* vault family small RNA gene (5q31.1), rs2811488 between the *C3orf27* and *RPN1* genes (3q21), and rs2302902 in the *ELK3* gene (12q23.1). We used Sanger bisulfite sequencing (bis-seq) of amplicons containing these SNPs and multiple (non-polymorphic) adjacent CpG dinucleotides to confirm ASM in heterozygous individuals in a panel of primary tissues chosen for relevance to complex diseases and representation of multiple cell lineages: peripheral blood (PBL), whole fetal and adult lung and adult bronchial epithelial cells, adult liver, adult brain (cerebral cortical grey matter), placenta (chorionic villi taken from near the fetal surface), human mammary epithelial cells (HMEC), and sperm. These analyses showed that the ASM was reproducible for all 4 index fragments, with the allelic asymmetry in methylation affecting multiple (non-polymorphic) CpGs around and including the index *Hpa*II sites (Fig. 1 and Figs. S2, S3, S4). Among these 4 regions, moderate ASM was observed in the *ELK3* gene, while the other index regions showed stronger allelic asymmetries. ASM was seen in two or more tissues for each of the index regions except the *C3orf27-RPN1* region, for which the index amplicon revealed highly tissue-specific ASM, which was very strong in the large majority of human placentas examined (33/34 cases; all tested using tissue from the fetal side of the organ, consisting of the free chorionic villi without maternal decidua; Table 1), but absent in the other tissues tested (Fig. 1, Table 1 and Figure S3).

**Table 1:** Summary of the 5 DMRs characterized in this report.

Chromosome position and index SNP	Gene(s)	Size of DMR	CTCF overlaps DMR?	Frequency and tissue-specificity of ASM^a^	Type of ASM
chr12:96,617,808 rs4762138 and chr12:96,617,304-96,617,304 rs2302902	*ELK3*	0.225 kb	NO	PBL (11/18), liver (1/11), HMEC (0/1)	Haplotype-dependent; rs4762138 A-allele hypermethylated, rs2302902 G-allele hypermethylated
chr2:120,048,479 rs1530562	*STEAP3* - *C2orf76*	0.45 kb	YES	PBL (20/29), liver (15/17), placenta (7/8), lung (3/4), HMEC (5/5), brain (5/5), sperm (0/1)	Haplotype-dependent; rs1530562 G-allele hypermethylated
chr19:41,386,494-41,386,494 rs3815710	*CYP2A7*	0.40 kb	YES	liver (8/10)	Haplotype-dependent; rs3815710 A- allele hypermethylated
chr3:128,336, 517 rs2811488	*C3orf27* - *RPN1*	1 kb	YES	PBL (0/12), liver (0/10), placenta (33/34), lung (0/3), HMEC (0/2), brain (0/4), sperm (0/2)	Imprinted; maternal allele hypermethylated^b^
chr5:135,415,476 rs2346019	*VTRNA2-1*	1.9 kb	YES	PBL (13/17), liver (17/17), placenta (11/15), lung (5/6), HMEC (3/3), brain (6/7), sperm (0/1)	Imprinted; maternal allele hypermethylated^c^

a Total number of samples with ASM/number of heterozygous samples tested, including Sanger and 454 long read bis-seq and *Hpa*II pre-digestion/PCR/RFLP assays.

b Maternal allele consistently hypermethylated; ASM restricted to the placenta; biallelic hypomethylation in other tissues examined, including sperm.

c Maternal allele consistently hypermethylated in all somatic tissues examined; biallelic hypomethylation in sperm.

### ASM in the *STEAP3-C2orf76* and *ELK3* regions is non-imprinted and haplotype dependent

We next asked whether the ASM in each of these 4 regions was due to parental imprinting or, alternatively, to *cis*-acting effects of the local DNA sequence or haplotype. Sanger and bis-seq in a total of 198 and 129 individuals with all 3 possible genotypes, plus *Hpa*II-pre-digestion/PCR/RFLP assays [7] in 76 and 30 heterozygotes, respectively, showed unequivocally that ASM both in the *STEAP3-C2orf76* intergenic region and in the *ELK3* intragenic region is haplotype-dependent: the simple genotypes at nearby SNPs consistently predicted the methylation status of 10 and 6 clustered CpGs, respectively, in these two index amplicons (Fig. 1 and Table 1). The ASM at both loci showed some tissue-specificity: for the *STEAP3*-*C2orf76* intergenic amplicon we found strong ASM in multiple tissues including brain, placenta, liver, HMEC, and PBL; with sperm DNA showing biallelic hypermethylation, while for the *ELK3* intergenic amplicon we found strong ASM in PBL and weak ASM in liver, with HMEC DNA showing biallelic hypermethylation (Fig. 1 and Table 1).

### ASM in the *VTRNA2-1* and *C3orf27*-*RPN1* regions is due to parental imprinting

**Figure 2:**
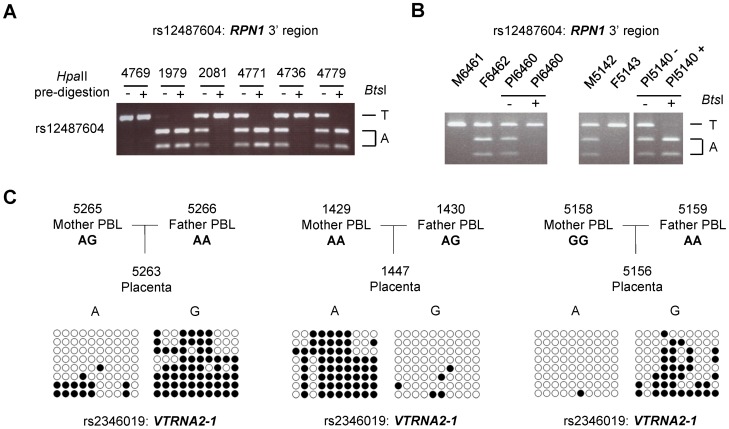
ASM downstream of the *RPN1* gene and in the *VTRNA2-1* gene is due to genomic imprinting with hypermethylation of the maternal allele. Trios consisting of matched parents (PBL) and offspring (placental chorionic villi from the fetal surface) were analyzed for ASM to determine the parental origin of the hypermethylated and hypomethylated alleles in the *C3orf27-RPN1* and *VTRNA2-1* index regions. **A,** *Hpa*II-predigestion/PCR/RFLP assay for ASM at non-polymorphic *Hpa*II sites in an amplicon including SNP rs12487604 (located immediately downstream of *RPN1*) which creates a *Bts*I restriction site. Undigested genomic DNA and genomic DNA pre-digested with the methylation-sensitive restriction enzyme *Hpa*II were amplified by PCR and then digested with *Bts*I. In the samples pre-digested with *Hpa*II (+) one allele drops out, indicating hypomethylation. A homozygous case for each allele is shown, as well as four heterozygotes; two cases show T-allele hypermethylation and two show A-allele hypermethylation. **B,** Trios analyzed by this assay showing parental imprinting of the index region downstream of *RPN1*. A total of 13 informative trios (two shown) were analyzed and each showed hypermethylation of the maternally-derived allele in the placenta (p<0.00311). **C,** Examples of bis-seq of trios for the index amplicon including SNP rs2346019 in the *VTRNA2-1* locus. The hypermethylated allele was found to be maternally derived in each of 10 informative trios (p<0.001565).

In contrast to the results for the *STEAP3-C2orf76* and *ELK3* index regions, Sanger and bis-seq in 192 and 325 individuals with all 3 possible genotypes and restriction pre-digestion/PCR/RFLP assays in 70 and 75 heterozygotes for the *C3orf27-RPN1* and *VTRNA2* index regions respectively showed that either allele could be relatively hypermethylated with the genotype of the index SNP having no predictive value - a situation suggestive of imprinting, in which methylation is dictated by parent-of-origin, not by haplotype (Table 1). To test for *bona fide* parental imprinting at these two loci we analyzed trios of maternal and paternal PBL and placenta DNA. In each of 13 trios informative for the *C3orf27-RPN1* index SNP and in each of 10 trios informative for the *VTRNA2-1* index SNP we found relative hypermethylation of the maternal allele in the placental DNA (p = 0.000311 for parental imprinting of the *C3orf27-RPN1* locus and p = 0.001565 for the *VTRNA2* locus, Chi-Square test; Fig. 2). While this manuscript was in preparation Treppendahl et al. reported ASM at *VTRNA2-1* in human hematopoietic cells [8] but imprinting was not tested in that study. Imprinting has not been previously reported in the *C3orf27*-*RPN1* region. As noted above, in the *RPN1* downstream DMR the ASM was restricted to placenta DNA samples, and in both loci sperm DNA showed biallelic hypomethylation, suggesting that the methylation imprint is acquired in the oocyte or early post-zygotically. The parent-of-origin dependence of the ASM in this region indicates that at least the initial “signal” for the imprint must be gametic, not somatic. However, we do not yet know the pattern of methylation in oocytes, so whether the densely methylated pattern of the maternal allele is established in the oocyte, or acquired early post-zygotically, remains to be determined.

By analogy with other imprinted genes [9], [10], the placenta-specific methylation imprinting of the *RPN1* 3′ region could be relevant to placental and fetal growth, so for planning future studies it is relevant to ask whether imprinting of this region also occurs in mice. The mouse genome shows conservation of synteny with regard to the linkage and order of the *Rab7-Rpn1-Gata2* genes, but an orthologue of human *C3orf27* (which is located between the human *RPN1* and *GATA2* genes) is not present in this region of the mouse genome. Moreover, the full-fledged CGI at the 3′ end of the *RPN1* gene in humans is not present in this position of the mouse *Rpn1* gene. Despite this lack of conservation of the CGI, and indeed the lack of conservation of the DNA sequence in this region, we tested for ASM in a CG-rich sequence located immediately downstream of *Rpn1* in the mouse, in a position analogous to the human DMR. Using extraembryonic tissues (yolk sac and placenta) from reciprocal inter-strain mouse crosses we found that CpG methylation in the orthologous region is somewhat allele-specific, but with much less allelic asymmetry than the human locus (Figure S5A). The methylation patterns in the mouse yolk sac and placenta are most simply explained by weak parental imprinting superimposed on a *cis*-acting haplotype effect. After accounting for the *cis*-effect (CAST allele more methylated than B6 allele), the direction of the weak imprinting matches that in humans, with the maternal allele relatively hypermethylated (Figure S5B). The main finding from this analysis - that the parental imprint is much weaker in mice - suggests that non-conserved, human-specific, sequences are important for establishing or stabilizing the methylation imprint in this chromosomal region.

A well-studied group of growth-regulating genes in fact show conserved imprinting in the placentas of humans and mice [10], [11], but the poor conservation of imprinting of the *RPN1* downstream region is not surprising, as gene regulation in the placenta is known to be rapidly and continually evolving in mammals along with changes in placental anatomy [12], [13]. Given that genomic imprinting appears to have evolved in mammals with placentation, a prediction is that the *C3orf27-RPN1* intergenic DMR, and its associated RNA transcripts, may play a “human-specific” role in placental growth and development that is not conserved in mice. Overall, these data add two new loci to the current list of approximately 80 human genes with robust methylation imprinting characterized to date.

### Discrete DMRs are found both in imprinted domains and in chromosomal regions with non-imprinted haplotype-dependent ASM

To compare the structures of chromosomal domains with imprinted versus non-imprinted ASM we used high throughput bisulfite PCR (Fluidigm AccessArray) with sample bar-coding, followed long-read 454 Pyrosequencing of amplicons bracketing 3 of the index regions; two with imprinted ASM (*VTRNA2-1* and *C3orf27*-*RPN1*) and one with non-imprinted/haplotype dependent ASM (*STEAP3*-*C2orf76*). To maximize the information from this approach we designed the amplicons to contain SNPs with heterozygosities ≥0.2 and to span DNA segments containing ≥4 CpG dinucleotides. For VTRNA2-1 region, 6 additional amplicons covering 3 CpGs were included. The long-read sequencing platform was optimal for analyzing ASM as it allowed us to capture the CpG methylation pattern and SNP genotype in each read, with no ambiguity as to phase, while the primer bar-coding strategy facilitated the analysis of a large series of individuals, with DNA samples from various tissue types including placenta, PBL, lung, liver, brain, heart, peripheral blood mononuclear cells (PBMC) and polymorphonuclear leukocytes (PMN).

**Figure 3:**
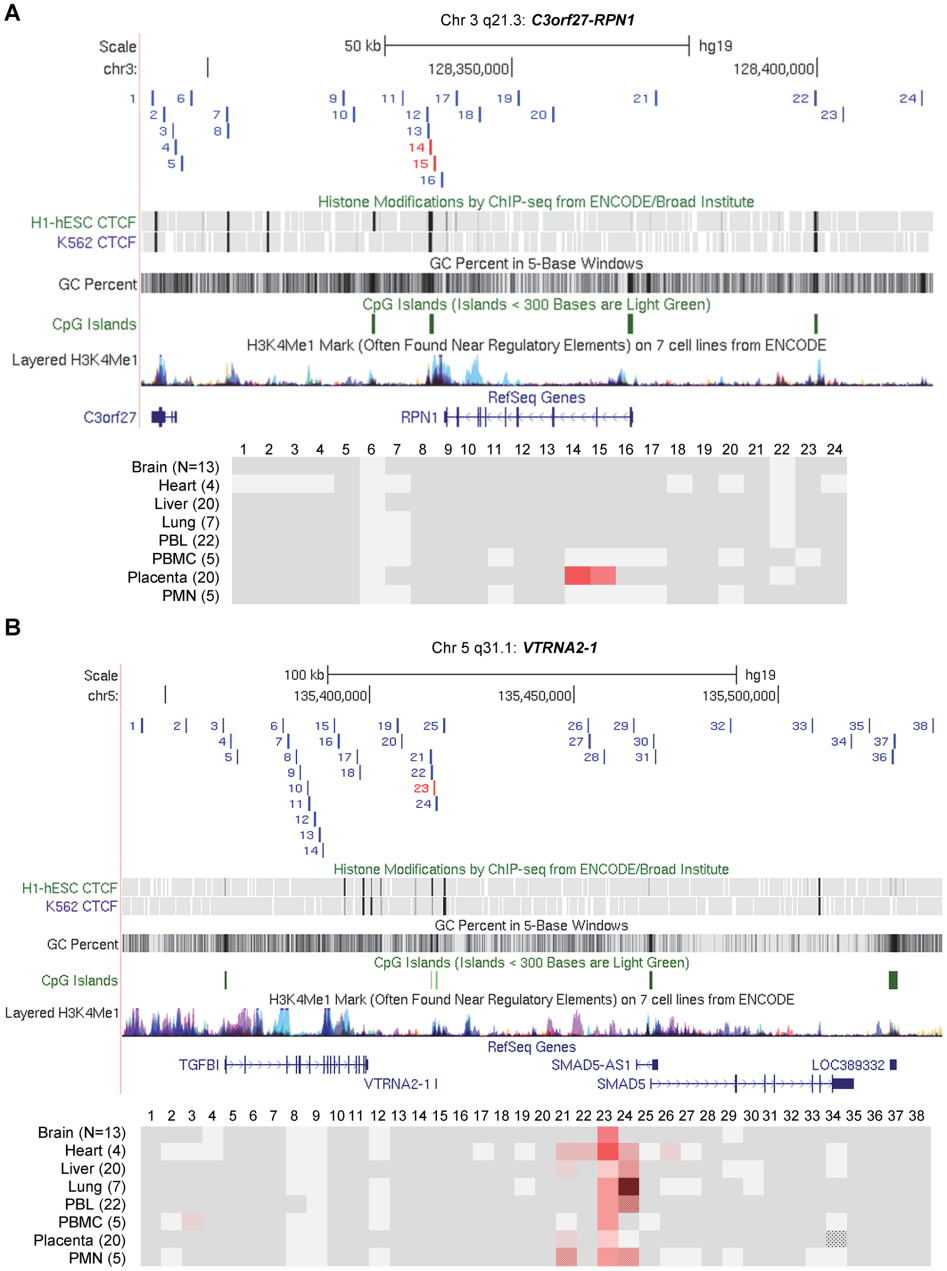
Long-range methylation mapping of the two imprinted regions, *C3orf27-RPN1* and *VTRNA2-1*, in 8 types of human tissues, shows small discrete DMRs. Maps from the UCSC genome browser (top) and ASM heat maps (bottom) derived from long read bis-seq in the gene regions. In the genome browser map, the index amplicons that provided the starting points for the mapping are in red, and CTCF binding sites, GC percentage, CGIs and H3K4me1 activating mark intensities are shown. In the heat maps, light grey shading indicates homozygosity in all samples, and dark grey indicates heterozygosity in one or more sample with no evidence of ASM. Shades of red indicate ASM, with the intensity scale indicating the absolute value of the difference in fractional methylation of the two alleles from 0.30 to 1. These data are averaged over all heterozygous samples for a given tissue. The red stippled cells indicate tissues with a greater than 0.30 average difference in fractional methylation of the two alleles *but* with only one heterozygous sample or with less than half of the samples showing significant ASM (p≤0.05, using the t-test). The grey stippled cell indicates an amplicon with insufficient read depth (<10). The number of samples subjected to bis-seq for each tissue is indicated in the parentheses. **A,** The *C3orf27-RPN1* region shows a single discrete epicenter of ASM (DMR) which overlaps with the initial index fragment discovered using MSNP data. ASM in this domain is only present in placenta. **B,** The *VTRNA2-1* region shows a single discrete epicenter of ASM (DMR) which overlaps with the initial index fragment discovered using MSNP data. ASM in this domain is present in all 8 tissues. However, ASM with less robust evidence and less consistency between samples (stippled red cells) was found in several immediate flanking amplicons (amplicons 21, 22, 24, 26).

**Figure 4:**
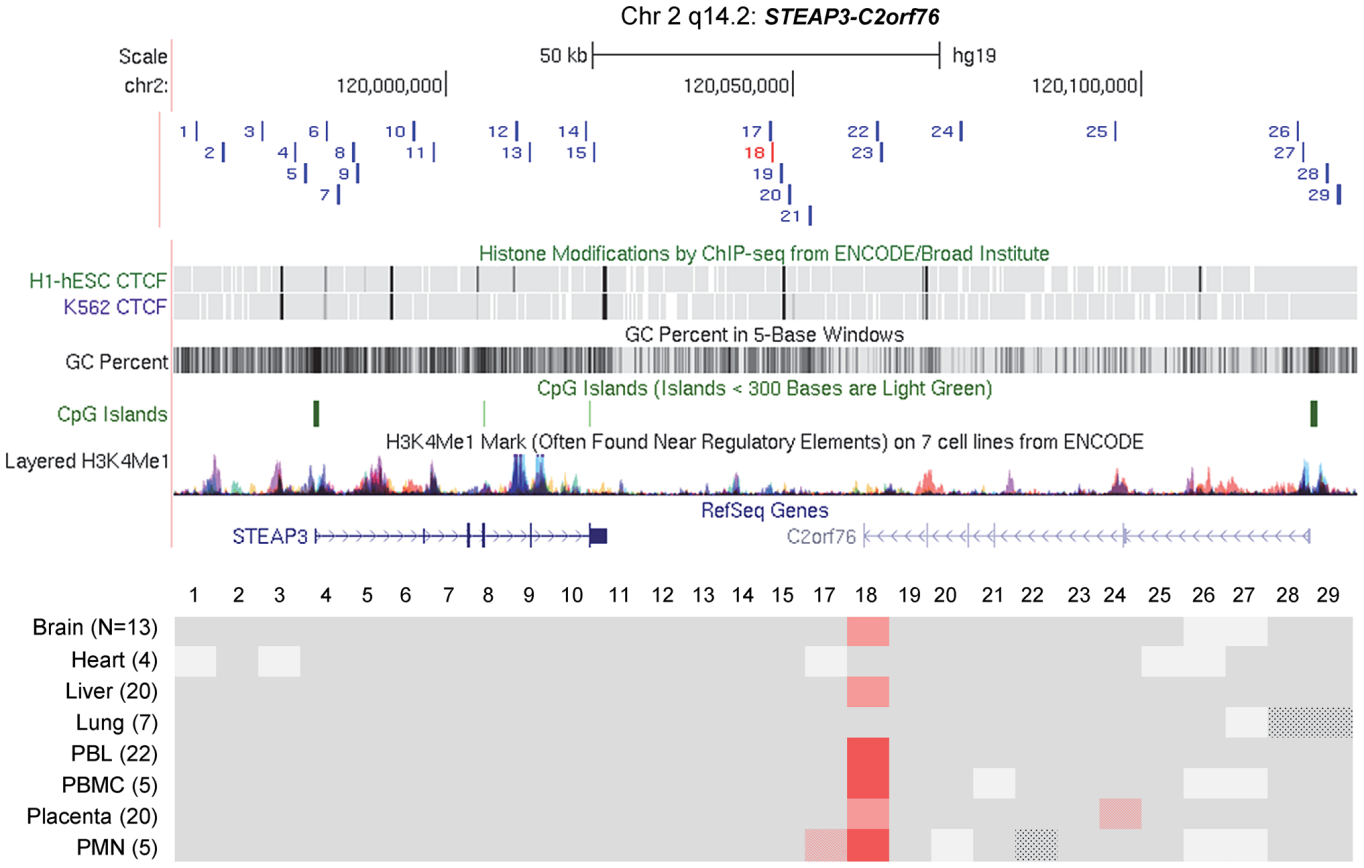
Long-range methylation mapping of the non-imprinted *STEAP3-C2orf76* region in 8 types of human tissues shows that small discrete DMRs can be present in loci with non-imprinted ASM. A map from the UCSC genome browser and an ASM heat map derived from long read bis-seq in the *STEAP3-C2orf76* region. In the genome browser map, the index amplicon that provided the starting point for the mapping is in red, and CTCF binding sites, GC percentage, CGIs and H3K4me1 activating mark intensities are shown. The presence and strength of ASM is color-coded as in Figure 3. This region shows a single discrete epicenter of ASM (DMR) which overlaps with the initial index fragment discovered using MSNP data. ASM in this domain is present in 6 tissues. The red stippled cells indicate tissues with a greater than 0.30 average difference in fractional methylation of the two alleles *but* with only one heterozygous sample or with less than half of the samples showing significant ASM (p≤0.05, using the t-test). The grey stippled cells indicate amplicons with insufficient read depth (<10).

The resulting dataset for the 3 chromosomal regions consisted of 104 amplicons analyzed in 96 biological samples, totaling 354 Mb of bis-seq, with a mean depth of 90 sequences per sample per amplicon and with a mean read length of 304 bp. Also included are data from >3500 full-length Sanger bis-seq reads of from 300 to 500 bp, which we carried out on multiple amplicons and tissue samples to supplement and verify the 454 data. Our strategy allowed us to score net and allele-specific methylation for each informative amplicon over multiple CpGs with complete genetic phase information in each read. The data are summarized by color-coding for ASM (difference in percent methylation of allele A versus allele B averaged for heterozygous samples by tissue) in Figure 3 and Figure 4. These “ASM heat maps” illustrate the amplicons for each chromosomal region, aligned to UCSC genome browser tracks [14], showing CpG islands (CGIs), histone modifications and CTCF binding sites.

**Figure 5:**
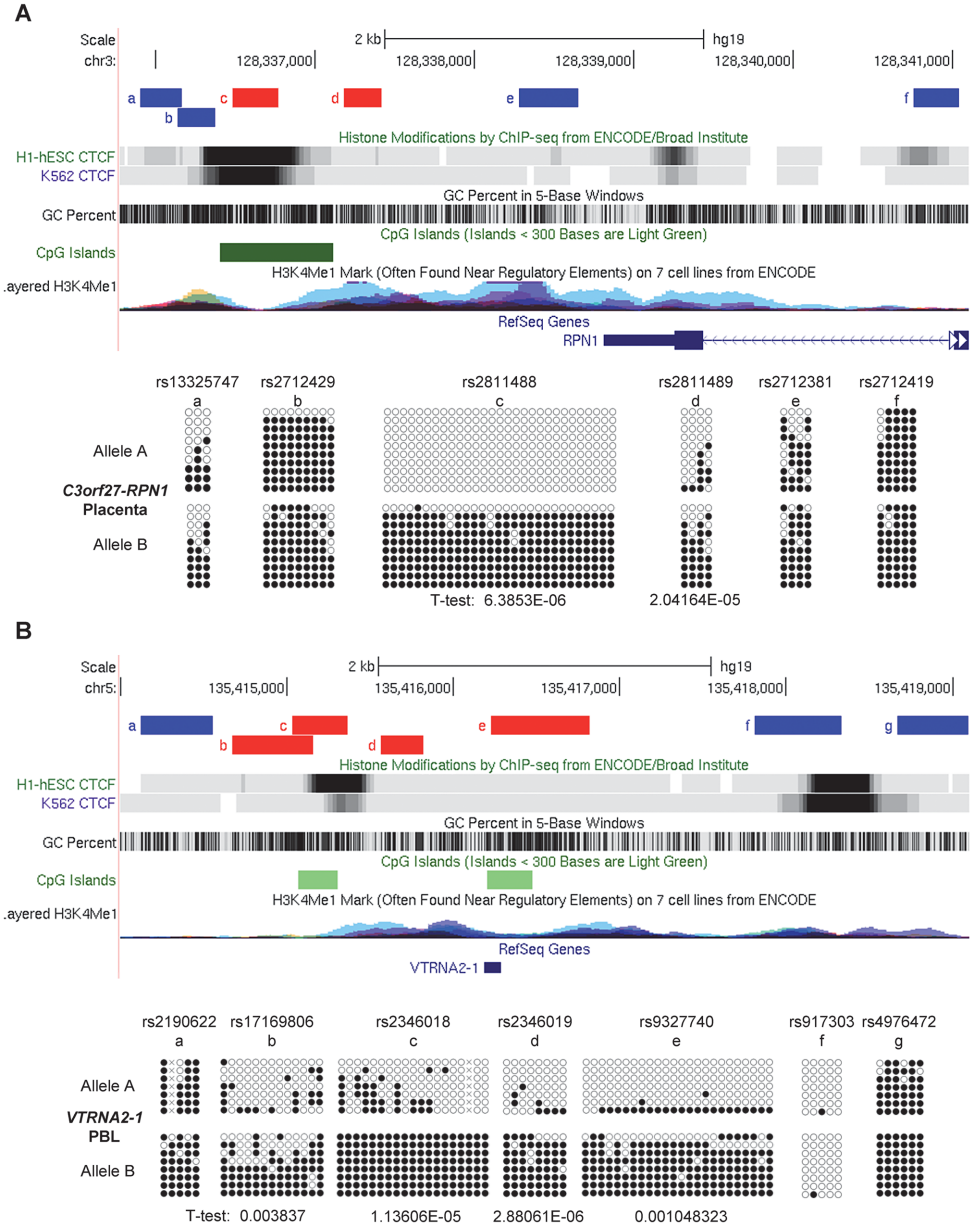
Local mapping of the imprinted *C3orf27-RPN1* and *VTRNA2-1* DMRs shows precise overlap with CTCF binding sites. **A,** Bis-seq of heterozygous placenta samples for amplicons immediately downstream of the *RPN1* gene shows ASM localized to a discrete region of about 1 kb in length (chr3: 128,336,485–128,337,414) spanning a strong CTCF binding site and a CGI. Amplicons with ASM are colored red. **B,** Bis-seq of heterozygous PBL samples for amplicons surrounding the *VTRNA2-1* vault RNA gene shows ASM localized to a 1.9 kb region (chr5: 135,414,670–135,416,821) spanning one CTCF binding site, two small CGIs, and the *VTRNA2-1* RNA gene, while another CTCF binding site upstream is hypomethylated. ASM for both genes was evaluated visually and by T-tests on the percent methylation of individual clones, comparing the sets of clones for the two alleles.

**Figure 6:**
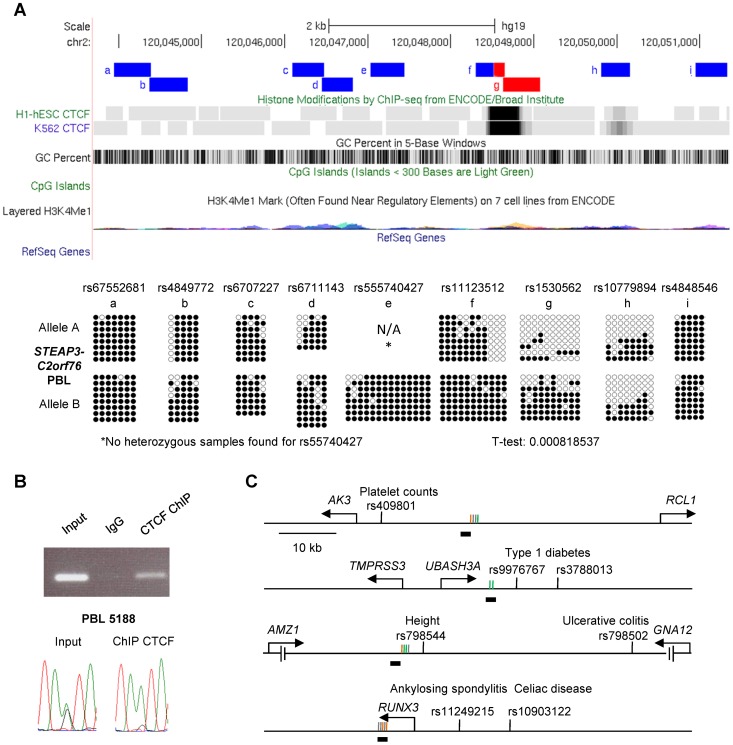
The *STEAP3-C2orf76* DMR with non-imprinted ASM overlaps a CTCF site, with CTCF preferentially bound to the unmethylated allele, and cross-indexing of ASM-associated CTCF sites with GWAS peaks provides evidence for regulatory haplotypes. **A,** Bis-seq of the *STEAP3 - C2orf76* intergenic DMR in heterozygous PBL samples shows localization of ASM to a discrete region about 450 bp in length (chr2: 120,048,634–120,049,081) spanning the strong CTCF binding site. *No heterozygous samples were found for rs55740427. **B,** PCR of the *STEAP3 - C2orf76* intergenic DMR performed on non-immunoprecipitated input DNA, negative control IgG IP DNA and CTCF ChIP DNA. As expected, the non-immunoprecipitated input sample results in a strong amplification while the negative control IgG IP sample shows no amplification. The observed amplification in the CTCF immunoprecipitated sample demonstrates CTCF binding at this region. Direct sequencing of end-point PCR products shows that the non-immunoprecipitated input DNA is heterozygous at the index SNP rs1530562 while the CTCF ChIP sample shows a strong enrichment for the A-allele indicating that CTCF is bound selectively to this (hypomethylated) allele. **C,** Maps of 4 representative haplotype blocks containing both a CTCF binding site with at least two ASM CpG sites (as defined by Chen et al. [15]) within a 1 kb window and SNPs associated with important human traits or disease susceptibility from GWAS (supra-threshold or strong sub-threshold peaks, all p<10^−6^; http://www.genome.gov/gwastudies). The relevant traits or diseases are indicated next to each SNP. The CpG sites that were analyzed for ASM (vertical lines) and located near the CTCF binding sites (horizontal black bars below the lines) are color-coded as orange for ASM sites that do not overlap with a CpG SNP, green for CpG SNP ASM sites, gray for non ASM sites. See also Table S4.

The most striking finding from this long-range analysis, and from additional intensive short-range mapping by Sanger bis-seq (Figs. 5 and 6A), is the discrete nature of all three DMRs; for all three regions, two with imprinted ASM and one with non-imprinted haplotype-dependent ASM, the allelic asymmetry in DNA methylation is restricted to one or two adjacent amplicons, with all three DMRs being less than 2 kb in length (Figs. 5 and 6 and Table 1). Within each of the regions examined, the flanking DNA outside of the DMRs showed varying levels of net CpG methylation, without asymmetry between the two alleles. Our mapping does not rule out additional DMRs farther away, but for the *STEAP3*-*C2orf76* region, which shows haplotype-dependent ASM, any DMRs farther away would be in separate haplotype blocks and therefore be independently regulated domains. We additionally carried out short-range mapping of ASM around the *ELK3* intragenic index fragment, which showed that it too is discrete and <2 kb in size (Figure S6).

### Both imprinted and non-imprinted DMRs can overlap precisely with CTCF binding sites

**Figure 7:**
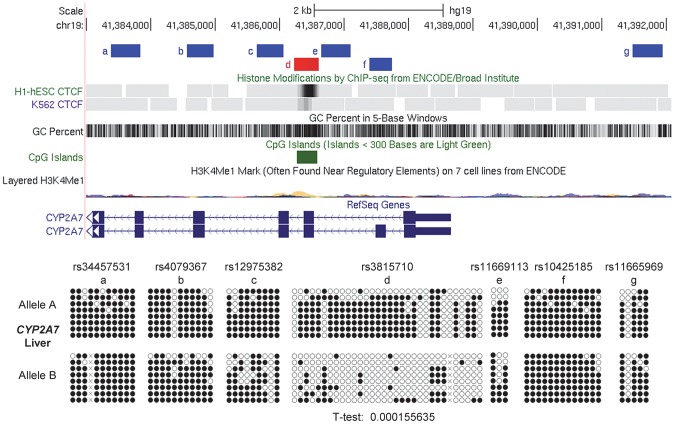
Local mapping of ASM in the *CYP2A7* gene shows a discrete DMR that precisely overlaps a CTCF binding site. Bis-seq of heterozygous liver samples for multiple *CYP2A7* amplicons shows ASM localized to a roughly 400 bp region (chr19: 41,386,227–41,386,613) spanning a CGI and a CTCF binding site in exon 2. ASM was evaluated visually and by a T-test on the percent methylation of individual clones, comparing the sets of clones for the two alleles.

Alignment of our methylation sequencing data with ChIP-Seq data from ENCODE and related projects, as displayed on the UCSC genome browser [14], showed that for both of the 2 imprinted domains, and for the non-imprinted *STEAP3*-*C2orf76* intergenic region, though not for the *ELK3* intragenic DMR, the DMRs with ASM overlapped precisely with empirically determined CTCF binding sites (Figs. 5, 6A and Figure S6). To ask whether there are additional examples of small discrete DMRs with strong and recurrent haplotype-dependent ASM that overlap with CTCF binding sites, we returned to an interesting locus with haplotype-dependent ASM and ASE that we had characterized in our initial report on this phenomenon [7], namely, the CYP2* gene cluster in chromosome band 19q13.2. As shown in Figure 7, the intragenic DMR in *CYP2A7*, tagged by SNP rs3815710, in fact shows discrete borders and precise co-localization with a CTCF binding site. Another DMR in this large gene cluster, tagged by SNP rs3844442 and located between 2 CYP2* pseudogenes [7], overlaps with a weaker CTCF binding site. Maps of each of these regions with haplotype-dependent ASM are shown aligned to haplotype blocks in Figure S7.

### Methylation-dependent binding of CTCF to the *STEAP3-C2orf76* DMR

To test directly for allele specific binding of CTCF to the *STEAP3-C2orf76* DMR, we carried out CTCF ChiP on a freshly obtained blood sample from a heterozygous individual that had shown strong sequence dependent ASM by previous bis-seq. By comparison with the IgG control IP, end-point PCR amplification of a region of the *STEAP3-C2orf76* DMR covering the index SNP rs1530562 confirmed CTCF binding to this region, and Sanger sequencing of the PCR product confirmed that only the hypomethylated allele was present in the CTCF enriched product, while both alleles were observed in the PCR product from the input non-immunoprecipitated DNA (Fig. 6B). Thus, CTCF binds specifically to the hypomethylated allele of the *STEAP3*-*C2orf76* intergenic DMR.

### Analysis of whole genome bis-seq data shows a non-random association of ASM sites with CTCF site locations and overlap with GWAS peaks

To test for a possible genome-wide non-random association of ASM with CTCF sites, we used previously published ASM site lists [15] from whole genome bis-seq of a single cell line, namely H1 hES cells [16]. Chen et al. [15] found 83,000 CpG sites with ASM, representing 14% of the total CpGs tested (a high percentage that partly reflects incomplete filtering-out of CpG SNPs in their analysis.) Based on their analysis, we first asked whether the ASM regions that we have described here were also detectable in H1 hES cells. The multi-tissue ASM we identified in the *STEAP3-C2orf76* and *VTRNA2-1* index regions was also present in the H1 hES cells, while the tissue-specific ASM found in *C3orf27-RPN1* was not present. In addition, the ASM we found in the *CYP2A7* region was not mirrored in H1 hES cells, consistent with tissue-specificity for liver.

We then looked for CTCF binding peaks in 500 bp windows centered on each of the CpGs tested by Chen et al. for ASM, using ChIP-seq data generated in H1 hES cells by the Broad/MGH, Hudson and UT-Austin ENCODE groups. We found 13,485 CTCF binding sites in the vicinity of the informative CpG sites. Most of these CTCF binding sites were in intragenic or intergenic regions (47%); fewer were in gene promoter regions (5%) or next to CGIs (7%). Overall, CTCF sites were far more frequently associated with unmethylated CpGs than with fully methylated, partially methylated or ASM CpGs (Figure S8). However, when considering only CpG sites in gene promoter regions (within 1 kb upstream and downstream of transcription starting sites), we find a 1.7-fold increase in the percent of ASM CpGs located near CTCF binding sites (that is, in a 500 bp window centered on the CpGs), compared to fully methylated CpG sites, but not unmethylated CpGs or partially methylated CpGs. When considering only the 99 ASM CpGs located in CGI-associated gene promoter regions (promoter CGIs) 40.4% were near CTCF sites, while less than 30% of fully methylated and partially methylated and only 23% of completely unmethylated CpGs in promoter CGIs were near CTCF sites (Figure S8A). Conversely, the distribution of the different classes of CpG sites (ASM, fully methylated, partially methylated, fully unmethylated) with the presence of CTCF binding sites in a 500 bp window centered on the CpG sites compared to their distribution without CTCF binding sites in such windows, reflected this association between ASM and CTCF binding sites in promoter regions and CGIs, with an increased proportion of ASM in these regions (Figure S8B).

Next, to more confidently enumerate the CTCF sites overlapping or very close to regions of ASM, we looked specifically for CTCF binding sites with ≥2 ASM CpGs (as defined by Chen et al. [15]) in a 1 kb window centered on the CTCF peak. A total of 158 CTCF sites met this criterion; among them, 142 were gene associated, with at least one named gene within 100 kb of the CTCF sites. Of these genes, we were able to cross-tabulate 75 to the current GWAS catalog (http://www.genome.gov/gwastudies). Interestingly, 61 of them were associated with a major human trait or disease susceptibility signal, and for 25 genes the CTCF binding sites, ASM and the GWAS peak SNP were all located in the same haplotype block - suggesting that these GWAS signals reflect a *bona fide* (functionally active) regulatory haplotype (Table S4, including our annotations for CpG SNPs, and examples in Fig. 6C). Thus, while CTCF sites are not enriched overall near regions of ASM, ASM-associated CTCF sites have a somewhat different distribution than other CTCF sites and, more importantly, are often associated with GWAS peaks for human traits and diseases.

### Allele-specific expression of some but not all genes near the imprinted and non-imprinted DMRs

**Figure 8:**
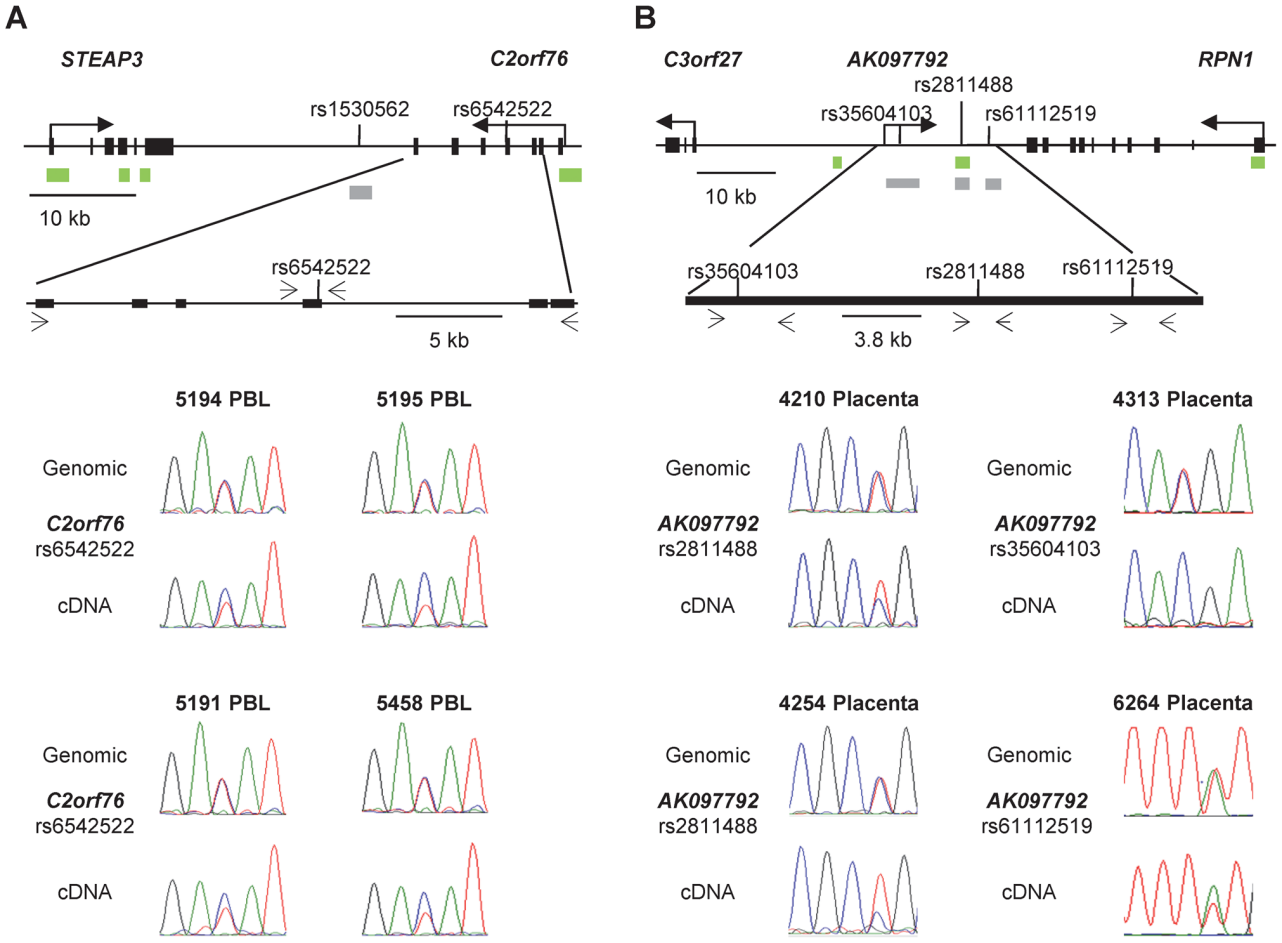
Non-imprinted ASM in the *STEAP3-C2orf76* region and imprinted ASM in the *RPN1-C3orf27* regions are both associated with ASE. **A,** ASE of the *C2orf76* gene, associated with the *C2orf76-STEAP3* DMR, is shown in duplicate for four PBL samples (rs6542522). In each case the C allele is preferentially expressed in the cDNA when compared to the genomic DNA, consistent with the sequence dependent nature of ASM in this locus. Overall, 12 informative PBL samples were analyzed in duplicate for ASE and 7 PBL samples showed preferential expression of the C allele. Likewise, preferential expression of the C allele was identified in 7 out of the 22 informative liver samples (data not shown). **B,** ASE of the lncRNA corresponding to the AK097792 EST, overlapping the index SNP rs2811488 in the *RPN1-C3orf27* intergenic DMR are shown on the left and those overlapping two additional informative SNPs, i.e. rs35604103 and rs61112519, are shown on the right for four placenta samples. Overall, the assays were performed in duplicates for 13 informative placenta samples. Ten of them showed a definite or complete ASE. In each of 6 informative samples assayed for both ASE and ASM, the hypermethylated maternal allele was the relatively repressed one.

One of the biologically important consequences of ASM is allele-specific RNA expression (ASE). We previously demonstrated strong haplotype-dependent ASE of the *CYP2A7* gene [7], and we were interested to ask whether ASE could be detected in the other chromosomal regions with ASM analyzed in this report. Assays comparing the representation of SNPs in genomic versus cDNA PCR products showed a definite and recurrent bias in allele-specific mRNA expression of the *C2orf76* gene, associated with the *STEAP3-C2orf76* DMR (Fig. 8). The untranslated AK097792 RNA transcript, associated with *RPN1-C3orf27* DMR also showed a recurrent bias in ASE: from 13 tested individuals informative for ASE, 6 showed a definite but not complete allele-specific bias, four had complete ASE and three were biallelically expressed (Fig. 8 and Fig. S3). In each of 6 informative samples with definite or complete ASE, and data for ASM, the relatively hypermethylated allele was the repressed one, suggesting a functional link between ASE and ASM at this locus. Interestingly, current ENCODE data support our findings from RT-PCR of a long non-coding RNA (lncRNA) traversing the DMR, and also suggest that a micro-RNA, as yet unnamed, arises from very close to this DMR (Figure S3).

The absence of transcribed SNPs prevented ASE assays for *VTRNA2-1*, but as was found by Treppendahl et al. [8] using a different cell line, our assays for activation of this gene by the demethylating drug 5aza-dC provided evidence for methylation-dependent expression of *VTRNA2-1* in HL60, ML3, and Jurkat leukemia cells (Figure S9). Additional analyses showed that the *STEAP3* gene is biallelically expressed in PBL and liver, while *TGFBI* and *SMAD5*, flanking *VTRNA2-1*, exhibit only a slight allelic expression bias in PBL, as do *RPN1* and *C3orf27* in placental samples (data not shown).

## Discussion

ASM is a well-studied hallmark of genomic imprinting. However, in the past several years this same type of allelic asymmetry has been found in and around many non-imprinted genes, both in normal human tissues and in F1 progeny of inter-strain mouse crosses [1], [17]. In contrast to imprinted ASM, in which the methylation status is dictated by the parent of origin of the allele, when ASM occurs at non-imprinted loci it is usually haplotype-dependent. Thus non-imprinted ASM is controlled by genetic polymorphisms, such as SNPs, indels and copy number variants (CNVs), which act in *cis* to set up the allelic asymmetries. The mechanism(s) by which this occurs are not yet understood. Research on this topic is important because haplotype-dependent ASM, with its functional correlate of genotype-specific gene expression manifesting as ASE and expression quantitative trait loci (eQTLs), is a major route by which inter-individual genetic differences in non-coding sequences can lead to differences in phenotypes, including disease susceptibility.

Here we have described new examples of genes and regulatory sequences with imprinted and non-imprinted ASM, which have revealed epigenomic features that are shared between these two epigenetic phenomena. Genes that are regulated by parental imprinting frequently have important roles in cell proliferation and tissue growth, so it is interesting that the VTRNA2-1 gene may have a suppressor role in human acute myeloid leukemias and other cancers [8], [18], and that vault RNAs are strongly upregulated during ES cell differentiation [19]. Similarly, given the clear importance of multiple imprinted genes in placental and fetal growth [10], [11], and the accumulating data for rare but recurrent alterations of maternal imprints in offspring conceived by assisted reproductive technologies, the imprinted *C3orf27-RPN1* chromosomal domain, including the lncRNA corresponding to the AK097792 EST, as well as other RNAs, such as the as yet unnamed micro-RNA precisely localized to this region by data from the ENCODE/CSH project (Figure S3) will be interesting for future studies of placental growth and function. The loci with non-imprinted ASM that we have described here are also potentially relevant to human traits. *CYP2A7* is located immediately adjacent to and is highly homologous to the *CYP2A6* gene, and both genes are predicted to encode cytochrome P450 proteins responsible for coumarin and nicotine metabolism and smoking behavior [20]. The *C2orf76* gene encodes a protein with unknown function, but a non-synonymous SNP in this gene was tentatively implicated in HIV susceptibility in African-Americans by GWAS at a nominal p-value of 0.0001 [21]. As we have previously pointed out [1], a major utility of mapping non-imprinted, haplotype-dependent ASM is to reveal *bona fide* regulatory haplotypes underlying human complex disease susceptibility. In this regard, our current analysis of prior whole genome bis-seq data has already yielded an interesting set of additional loci in which GWAS signals for important human traits and disease susceptibility coincide with both ASM and CTCF sites.

Lastly, our data lay an essential groundwork for testing mechanisms of haplotype-dependent ASM. One feasible application will be in determining the minimal sequence requirements that allow local haplotypes to dictate methylation patterns in *cis*: based on our mapping data it should be possible to design allelic series of large bacterial artificial chromosomes (BACs) containing the chromosomal regions described here, which can be subjected to targeted site-directed mutagenesis, including the CTCF sites as well as haplotype-specific sequences outside of these sites, and then analyzed for *cis*-effects on CpG methylation patterns in vivo in BAC-transgenic mice.

## Materials and Methods

### MSNP analysis

Tissues from human organs were obtained, without patient identifiers, from the Molecular Pathology Shared Resource of the Herbert Irving Comprehensive Cancer Center and blood samples from volunteers were obtained with informed consent under protocols approved by the internal review board of Columbia University Medical Center. MSNP assays were performed as previously described [7]. Affymetrix 250K *Sty*I and 6.0 SNP arrays were used, with initial complete digestions of the genomic DNA samples with *Sty*I (250K arrays) or *Nsp*I (6.0 arrays), with the methylation-sensitive restriction enzyme *Hpa*II or with its methylation-insensitive isoschizomer *Msp*I, followed by probe synthesis and labeling as recommended by Affymetrix. The MSNP data were processed numerically as described [7], followed by visualization of the allele-specific hybridization data in dChip [22] to identify SNP-tagged *Sty*I or *Nsp*I restriction fragments where call conversions were made from AB with no *Hpa*II to AA or BB with *Hpa*II pre-digestion. Based on recurrent call conversions in two or more samples and consistency of hybridization across multiple probes on visualization of the intensity data, we selected loci for validation of ASM by bis-seq of amplicons spanning the original index SNPs.

### Local methylation mapping by Sanger bis-seq

We used Sanger bis-seq of multiple clones for intensive short-range mapping of ASM in each of the 5 chromosomal regions, as well as for filling gaps in the long-read 454 bis-seq. Genomic DNA (0.5 µg) was bisulfite converted using the EpiTect Kit (Qiagen) following the manufacturer's protocol. Primers were designed in MethPrimer [23] to encompass >5 contiguous CpGs and one or more SNP, and the converted DNA was amplified for regions of interest using optimized PCR conditions (Table S1). PCR reactions were performed in duplicate then pooled and purified using the Wizard SV System (Promega), followed by cloning using the TOPO TA Kit (Invitrogen). Multiple clones were sequenced.

### Extended methylation mapping by Fluidigm bisulfite PCR and 454 long-read sequencing

Primer sets spanning 50–100 kb on each side of each of the three index fragments for long-range mapping were designed as described in [24] and in part empirically, using both strands of an *in silico* bisulfite-converted SNP-masked genome. Primer sequences were not allowed to overlap with any SNPs having >1 percent heterozygote frequency in dbSNP130. Amplicons were allowed to range between 200–650 bp in size, with most between 200–450 bp. CpG content was allowed to range between 0–1 CpGs per primer with zero CpGs preferred. T_m_ was allowed to range from 56–60°C, primer GC content is allowed to range between 35–65%, and self-complementarity, primer-primer complementarity, and hairpin formation is restricted for stable structures with melting temperatures above 46°C. Primers generating off target amplicons of size 50–2000 bp (with two mismatches allowed) were filtered out by scanning against a bisulfite converted genome with NCBI reverse e-PCR. From these primer sets, 41 amplicons for the *VTRNA2-1* region, 27 amplicons for the *STEAP3*-*C2orf76* region and 25 amplicons for the *C3orf27-RPN1* region were picked based on minimum cut-offs for the number of CpG sites encompassed in each amplicon (≥4), and heterozygosity frequencies of annotated SNPs between the primers (≥.2). For VTRNA2-1 region, 6 additional amplicons covering 3 CpGs were included. Primer sequences are in Table S2. Bisulfite modification was performed for 96 samples using 2 ug of genomic DNA and the Epitect 96 Kit (Qiagen), following the recommended protocol. The bisulfite modified DNA was re-precipitated and concentrated using Pellet Paint (EMD Chemicals). To amplify the DNA from the 96 samples, the Fluidigm 48×48 Access Array was used, with JumpStart Taq Polymerase (Sigma) and a 60°C touchdown for ten cycles followed by 51°C for 29 cycles of amplification. Of the resulting PCR products, one microliter of a 1∶50 dilution was used in a second barcoding PCR reaction. Fluidigm barcodes designed with 454 adapters were added to samples 1–48 and 49–96 during this PCR step. Both the 454 FLX instrument and 454Junior instrument and their emPCR and Sequencing kits (Roche) were used. The barcoded PCR products were pooled and purified using Agencourt AMPure XP beads and a titration was performed (described in 454 provided protocol) in order to find the best bead∶DNA ratio required to remove DNA fragments smaller than 300 bp. The purified PCR products were then prepared for sequencing following the 454 emulsion PCR protocol (Roche). A titration of bead∶DNA ratios was also performed during this step to ensure an ideal ratio was achieved in order to obtain the best sequencing results. The amplified PCR products were then sequenced using the sequencing kits designed for each instrument. The filter settings were adjusted on the 454 sequence analysis software to a vfTrimBack Scale Factor of .5. The sequences were then mapped, scored for each previously identified SNP, and each allele was analyzed for percent methylation, both at individual CpGs and averaged over all CpGs in the amplicon. Of the original 99 primer pairs, 91 gave a sufficient number of reads to allow genotyping (minimum ten reads with at least 20% of reads representing the minor allele), and net methylation analysis in most samples, and in at least 3 samples per tissue (minimum 7 reads), as well as scoring ASM in multiple heterozygous samples. Also included were data from >3500 full-length Sanger bis-seq reads of from 300 to 500 bp performed on several amplicons and tissues. T-tests were performed to determine significant ASM, p<0.05, for samples with at least 5 reads per allele.

### Q-PCR and analysis of allele specific mRNA expression (ASE)

Tissue and blood cell RNA was extracted using Trizol reagent (Invitrogen) following the manufacturer's protocol. The RNA was then reverse transcribed to cDNA using RT Kit (AmbionRETROscript) with random hexamer primers. PCR primers were designed for genomic DNA and cDNA to cover a SNP in the exon of the gene of interest using Primer3 software, with the cDNA primers spanning an intron of the gene, or in the case of the AK097792 non-translated RNA, not spanning an intron but verified as amplifying only cDNA in the samples tested, by lack of PCR product in the minus-RT control reaction (Table S3). Genomic DNA and cDNA were amplified by PCR and the PCR products were Sanger sequenced from duplicate reactions. The sequence chromatograms were analyzed by comparing peak heights of the two alleles in heterozygous individuals in the genomic DNA versus cDNA. For measuring expression of the *VTRNA2-1* small RNA we used a custom TaqMan assay (Applied Biosystems) according to the recommended protocol.

### Chromatin immunoprecipitation (ChIP)

Peripheral blood mononuclear cells (PBMCs) were isolated from whole blood following the manufacturer's instructions for FicollPaque Plus reagent (GE). Chromatin Immunoprecipitation was performed on the PBMCs using the Magna-ChIP G Kit from Millipore following the suggested protocol with some slight modifications. Cell fixation was performed for six minutes. Sonication was performed using a Fisher Sonic Dismembrator at a power setting of 3.2 for 2 minutes total sonication time with 2 seconds sonication followed by 8 seconds recovery. Four micrograms of anti-CTCF antibody (Millipore) was used in an overnight incubation at 4°C with sheared chromatin and protein G Magna ChIP beads. PCR for the *STEAP3-C2orf76* DMR was then performed using primers: Forward, GACAGACTCTGCTGCCACCT and Reverse, AGCAGCTTCTTCTCGGTATG. Two microliters of purified input, negative control IgG and CTCF immunoprecipitated samples were used for PCR with a touchdown from 60°C to 51°C for 38 cycles.

### Analysis of genome-wide association of ASM with CTCF binding sites

Previously published ASM site lists from the whole genome bis-seq of H1 hESC cells were downloaded [15], [16]. The dataset included 514,181 CpG sites tested for ASM by Chen et al. [15]. Of these, 79,365 were CpG sites classified as ASM. For this analysis, in addition to ASM sites, we grouped CpG sites without evidence of ASM into 3 categories: full methylation (200,318 CpG sites with >90% of reads with methylation at the CpG site), partial methylation (232,724 CpG sites with 10% to 90% of reads with methylation at the CpG site) or complete lack of methylation (1,774 CpG sites with <10% of reads with methylation at the CpG site). To assess the presence of CTCF binding peaks in a 500 base window centered on each CG tested for ASM, we used ChIP-seq data generated in H1 hESC cells by the Broad/MGH (UCSC accession number: wgEncodeEH000085), Hudson alpha (UCSC accession number: wgEncodeEH001649) and UT-Austin (UCSC accession number: wgEncodeEH000560) ENCODE groups. We searched for CTCF binding sites present in at least one of these datasets and found 13,485 CTCF binding sites in the vicinity of the CpG sites included into this analysis. We performed sub-analyses in order to look specifically at CpG sites in intergenic regions or intragenic region, as well as in gene promoter regions (1 kb upstream and downstream of transcription starting sites of genes) and/or close to CGIs (within 250 bp), using data available from the UCSC genome browser. All p-values for these analyses were calculated using one-sided Fischer exact tests.

## Supporting Information

Figure S1 Primary MSNP data for the index regions in this study. MSNP data (allele-specific hybridization intensities from Affymetrix SNP 250K StyI and SNP 6.0 arrays) were processed in dChip as in Kerkel et al., 2008. For each of the index regions shown, the heterozygous SNP call changes to a “homozygous” call when the genomic samples are digested with the methylation-sensitive restriction enzyme *Hpa*II prior to probe synthesis. The index amplicons for verifying ASM are indicated by the grey rectangles below the maps; CG-islands (CGIs) are indicated by green rectangles. **A,** Primary MSNP data for the *ELK3* index region. **B,** Primary MSNP data for the *C3orf27-RPN1* index region. **C,** Primary MSNP data for the *VTRNA2-1* index region. **D,** Primary MSNP data for the *STEAP3-C2orf76* index region. The index region between *STEAP3* and *C2orf76* was identified initially using an acute myeloid leukemia (AML) sample, but was then validated by pre-digestion/PCR and bis-seq data as showing ASM in numerous normal human tissue samples.
(TIFF)

Figure S2 Examples of additional biological samples with non-imprinted haplotype-dependent ASM in the *STEAP3-C2orf76* intergenic region. The ASM is strongest in PBL and acute myeloid leukemia cases (AML). There is variable ASM in the liver samples and weaker ASM in the HMEC samples. In all cases the G-allele is relatively hypermethylated.
(TIFF)

Figure S3 Bisulfite sequencing of heterozygous and homozygous samples in the imprinted index region immediately downstream of the *RPN1* gene. LncRNA mapping in *C3orf27-RPN1*. **A,** Homozygous and heterozygous placenta, sperm and human mammary epithelial cell (HMEC) samples are shown. The two homozygous placentas (upper-left panel) show a biphasic methylation pattern consistent with imprinting. The four heterozygous placental samples show strong ASM with the G allele or A allele hypermethylated, also consistent with imprinting (upper-right and middle panel). The parent-of-origin dependence of the ASM, that is, proof of imprinting, is shown in Figure 2 of the main text. No methylation is seen in the HMEC and sperm samples (lower panel). **B,** LncRNA in *C3orf27-RPN1* region. We used RT-PCR of DNAse-treated placental RNA samples to map a minimum region over which the lncRNA is detectable. The black solid lines show the position of the amplicons used and the lncRNA transcript is indicating with (+) on the top of the amplicons. A graphical representation, based on the available data from the USCS genome browser, for human ESTs, long RNA-seq and small RNA-seq is shown below the map.
(TIFF)

Figure S4 Examples of additional biological samples with ASM in the imprinted *VTRNA2*-1 gene. Strong ASM is seen in lung, HMEC and liver samples, while the sperm samples are unmethylated.
(TIFF)

Figure S5 CpG methylation patterns in the mouse *C3orf27-Rpn1* region are determined by a cis-acting haplotype effect superimposed on weak parental imprinting. **A,** Map of the region of the mouse genome that is orthologous to the human *C3orf27-RPN1* region and bisulfite sequencing of the *Rpn1* downstream region. There is only partial conservation of synteny. Primers used for bisulfite PCR were GGGTTAAGGGATTGTTTAAATAGTTA and ACCAAACCTTTAAACCAAAAAAAAC, which amplify a 390 bp CG-rich sequence that does not meet formal criteria for a CGI. **B,** Percent methylation of each allele as a function of strain-of-origin and parent-of-origin. T-tests were performed on the dataset consisting of the methylation values for each of the allele-specific bisulfite clones in each of the indicated yolk sac samples with known strain- and parent-of-origin.
(TIFF)

Figure S6 Local methylation mapping of the *ELK3* DMR. Bisulfite sequencing of heterozygous PBL samples for multiple *ELK3* amplicons shows the specific range of sequence dependent ASM in this locus, spanning 225 bp (chr12: 96,617,249–96,617,474).
(TIFF)

Figure S7 Haplotype blocks aligned to the loci with non-imprinted sequence dependent ASM using International HapMap Project (http://hapmap.ncbi.nlm.nih.gov/). In the gene maps the grey bars indicate the index DMRs, green bars indicate CGIs, blue bars are CTCF sites and the black rectangles are gene exons. Each of the DMRs is within a block of strong linkage disequilibrium. The *STEAP3-C2orf76* DMR (A) and the *CYP2A7* DMR (C) overlap CTCF binding sites, while the *ELK3* DMR (B) , which has recurrent but less strong ASM, does not.
(TIFF)

Figure S8 Association of ASM CpGs with particular locations of CTCF binding sites in H1-hESC. **A,** Bar graphs and table showing the percentage of the different classes of CpG sites in or near CTCF binding sites (in 500 bp windows centered on the CpG sites analyzed by Chen et al. [15]) in H1-ES cells. Classes of CpG sites (ASM as determined by Chen et al. [15], or fully, partially, or unmethylated, as determined by us) are color coded. Separate analyses have been performed for CpG sites in intergenic regions, intragenic regions, promoter regions, CGIs and promoter CGIs. The total numbers of sites and one sided Fischer exact p-values for the enrichment of CTCF sites, compared to CpGs that are fully methylated, partially methylated or fully unmethylated, are indicated in the table. **B,** Bar graphs showing the distribution of the different classes of CpG sites (ASM, fully methylated, partially methylated, fully unmethylated), with or without a CTCF binding site in a 500 bp window centered on the CpG. The same color codes as in panel A are used for ASM, fully methylated, partially methylated and fully unmethylated CpG sites. Separate analyses have been performed for CpG sites in intergenic regions, intragenic regions, promoter regions, CGIs and promoter CGIs.
(TIFF)

Figure S9 Effects of the demethylating drug 5aza-dC on expression of *VTRNA2-1* RNA in HL60, ML3 and Jurkat cell lines. In each cell line, cells were treated with the indicated concentrations for three days. Increased expression of VTRNA2 is observed in all three cell lines and a reduction in methylation was confirmed in Jurkat cells for the index region DMR (data not shown).
(TIFF)

Table S1 PCR primers for local mapping of ASM by Sanger bis-seq.
(XLSX)

Table S2 PCR primers for regional mapping by Fluidigm bisulfite PCR followed by 454 sequencing.
(XLSX)

Table S3 PCR primers for assessing ASE by cDNA/gDNA sequencing.
(XLSX)

Table S4 Loci with ASM CpGs (>2) near CTCF sites: cross-tabulated to GWAS peaks. This table is from our analysis of the original whole genome bis-seq data of Lister et al. (2009) Human DNA methylomes at base resolution show widespread epigenomic differences( Nature 462: 315–322), as interpreted for ASM by Chen et al. (2011, A comparative analysis of DNA methylation across human embryonic stem cell lines. Genome Biol 12: R62.).
(XLSX)
