# Supplementary figures and images for: Comparative Anatomy of Chromosomal Domains with Imprinted and Non-Imprinted Allele-Specific DNA Methylation
Source: PLoS Genet. 2013 Aug 29;9(8):e1003622. doi: 10.1371/journal.pgen.1003622 (PMC3757050; doi:10.1371/journal.pgen.1003622)

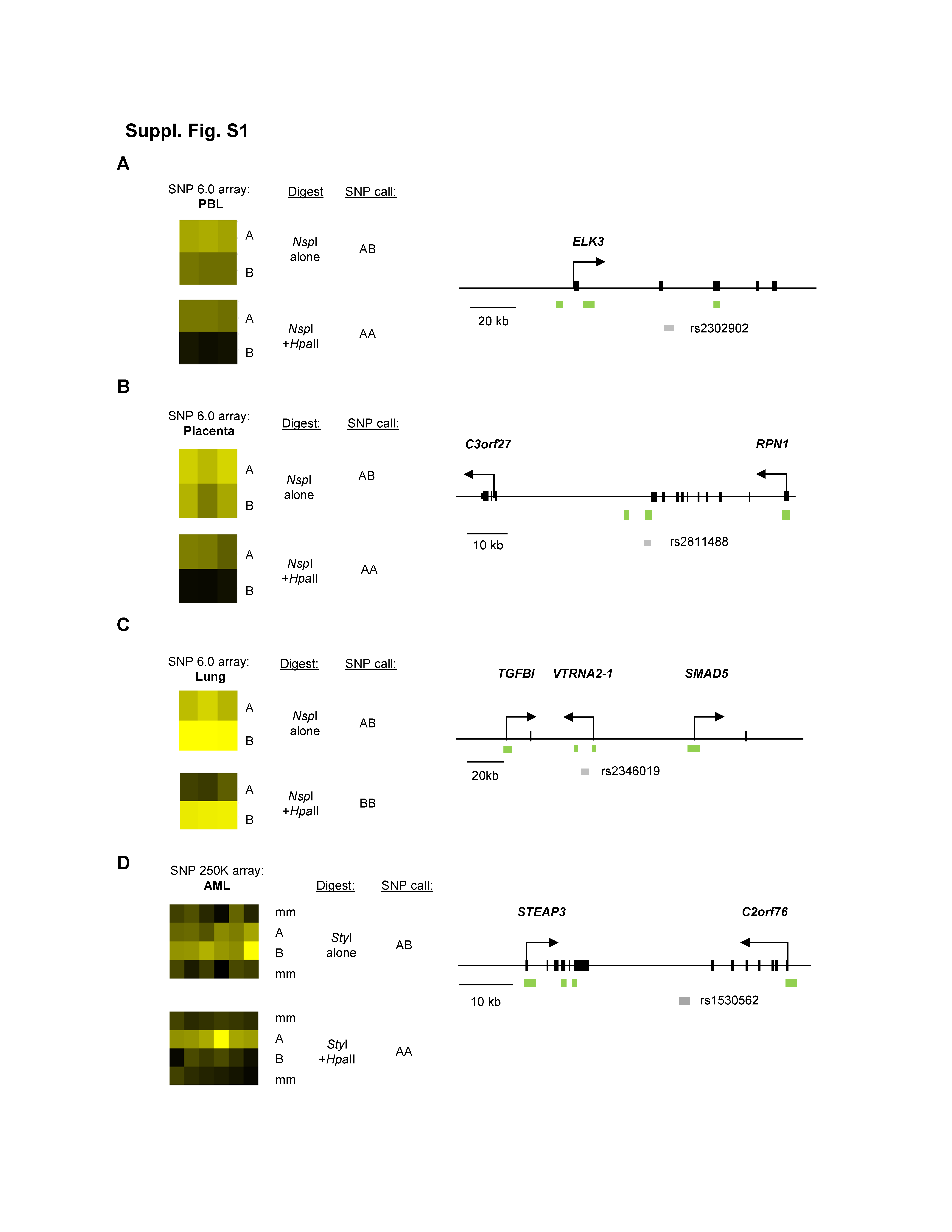

Supplement: Figure S1 — Primary MSNP data for the index regions in this study. MSNP data (allele-specific hybridization intensities from Affymetrix SNP 250K StyI and SNP 6.0 arrays) were processed in dChip as in Kerkel et al., 2008. For each of the index regions shown, the heterozygous SNP call changes to a “homozygous” call when the genomic samples are digested with the methylation-sensitive restriction enzyme HpaII prior to probe synthesis. The index amplicons for verifying ASM are indicated by the grey rectangles below the maps; CG-islands (CGIs) are indicated by green rectangles. A, Primary MSNP data for the ELK3 index region. B, Primary MSNP data for the C3orf27-RPN1 index region. C, Primary MSNP data for the VTRNA2-1 index region. D, Primary MSNP data for the STEAP3-C2orf76 index region. The index region between STEAP3 and C2orf76 was identified initially using an acute myeloid leukemia (AML) sample, but was then validated by pre-digestion/PCR and bis-seq data as showing ASM in numerous normal human tissue samples. (TIFF) [file pgen.1003622.s001.tiff]

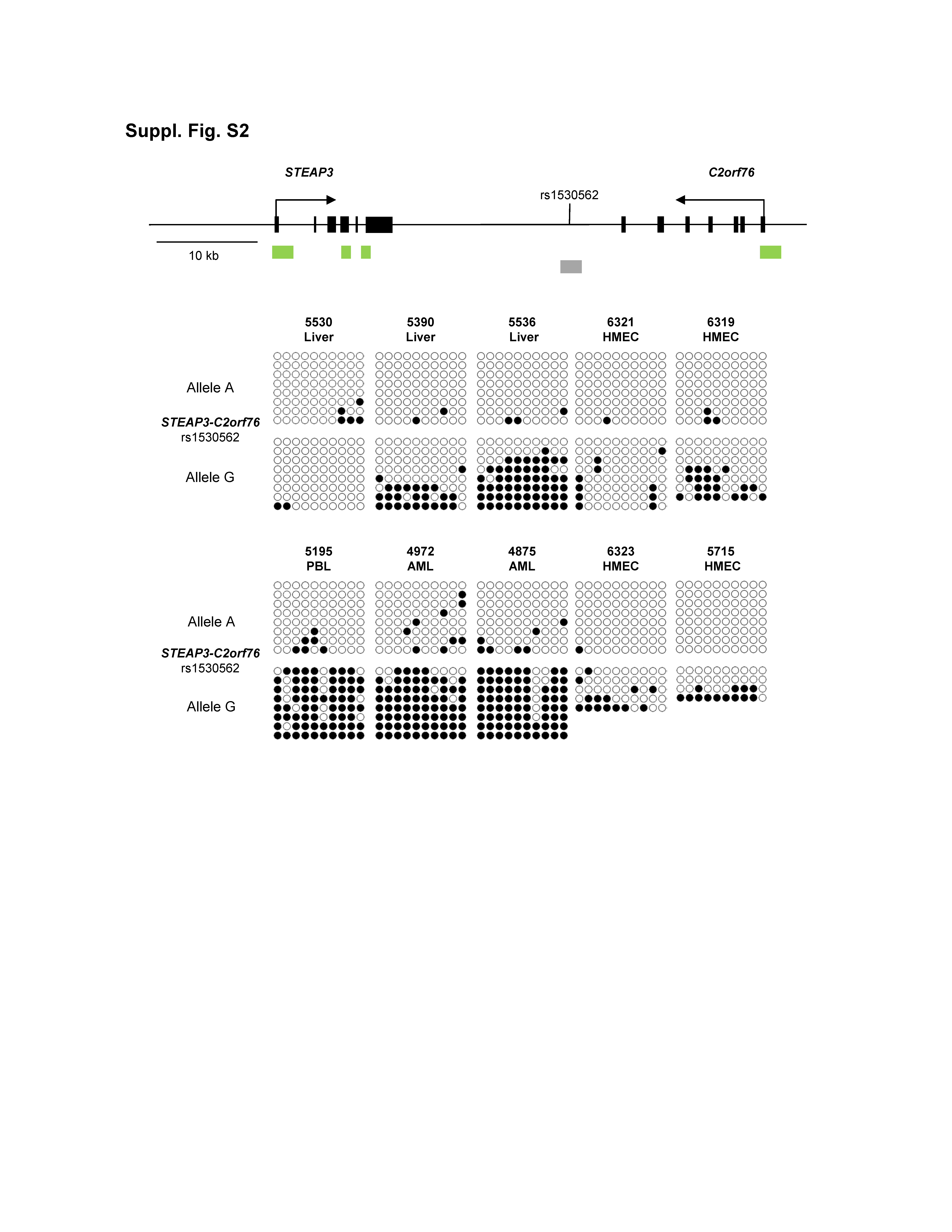

Supplement: Figure S2 — Examples of additional biological samples with non-imprinted haplotype-dependent ASM in the STEAP3-C2orf76 intergenic region. The ASM is strongest in PBL and acute myeloid leukemia cases (AML). There is variable ASM in the liver samples and weaker ASM in the HMEC samples. In all cases the G-allele is relatively hypermethylated. (TIFF) [file pgen.1003622.s002.tiff]

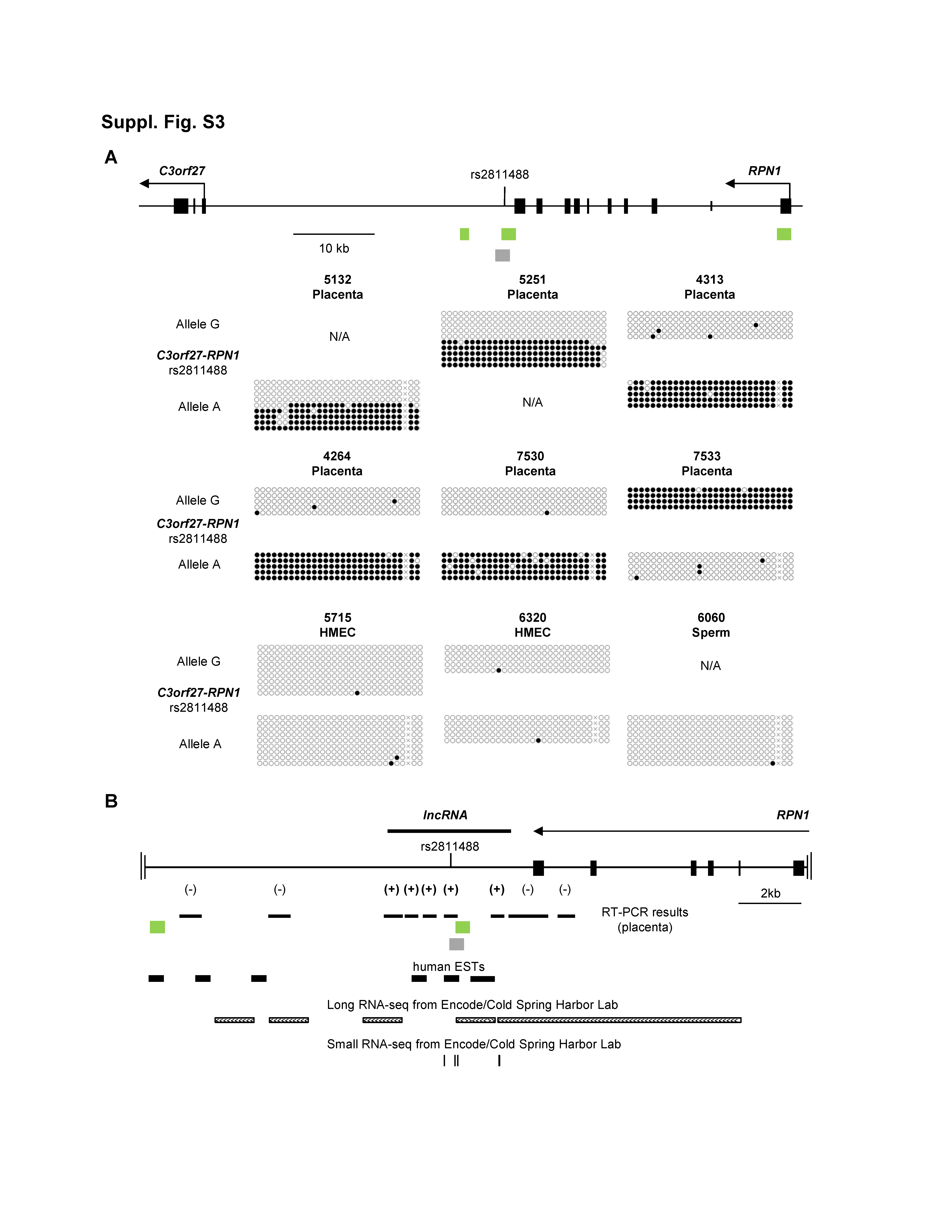

Supplement: Figure S3 — Bisulfite sequencing of heterozygous and homozygous samples in the imprinted index region immediately downstream of the RPN1 gene. LncRNA mapping in C3orf27-RPN1. A, Homozygous and heterozygous placenta, sperm and human mammary epithelial cell (HMEC) samples are shown. The two homozygous placentas (upper-left panel) show a biphasic methylation pattern consistent with imprinting. The four heterozygous placental samples show strong ASM with the G allele or A allele hypermethylated, also consistent with imprinting (upper-right and middle panel). The parent-of-origin dependence of the ASM, that is, proof of imprinting, is shown in Figure 2 of the main text. No methylation is seen in the HMEC and sperm samples (lower panel). B, LncRNA in C3orf27-RPN1 region. We used RT-PCR of DNAse-treated placental RNA samples to map a minimum region over which the lncRNA is detectable. The black solid lines show the position of the amplicons used and the lncRNA transcript is indicating with (+) on the top of the amplicons. A graphical representation, based on the available data from the USCS genome browser, for human ESTs, long RNA-seq and small RNA-seq is shown below the map. (TIFF) [file pgen.1003622.s003.tiff]

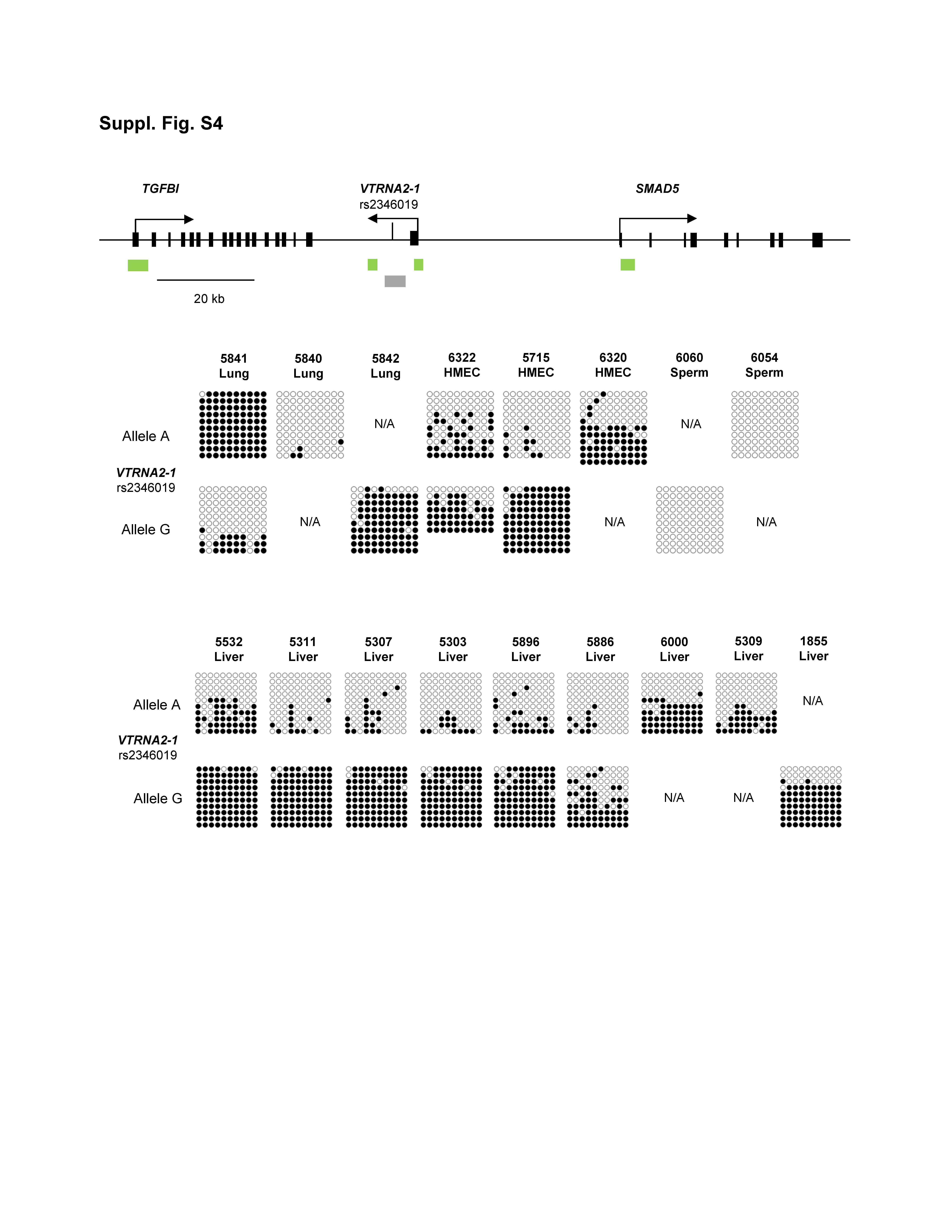

Supplement: Figure S4 — Examples of additional biological samples with ASM in the imprinted VTRNA2-1 gene. Strong ASM is seen in lung, HMEC and liver samples, while the sperm samples are unmethylated. (TIFF) [file pgen.1003622.s004.tiff]

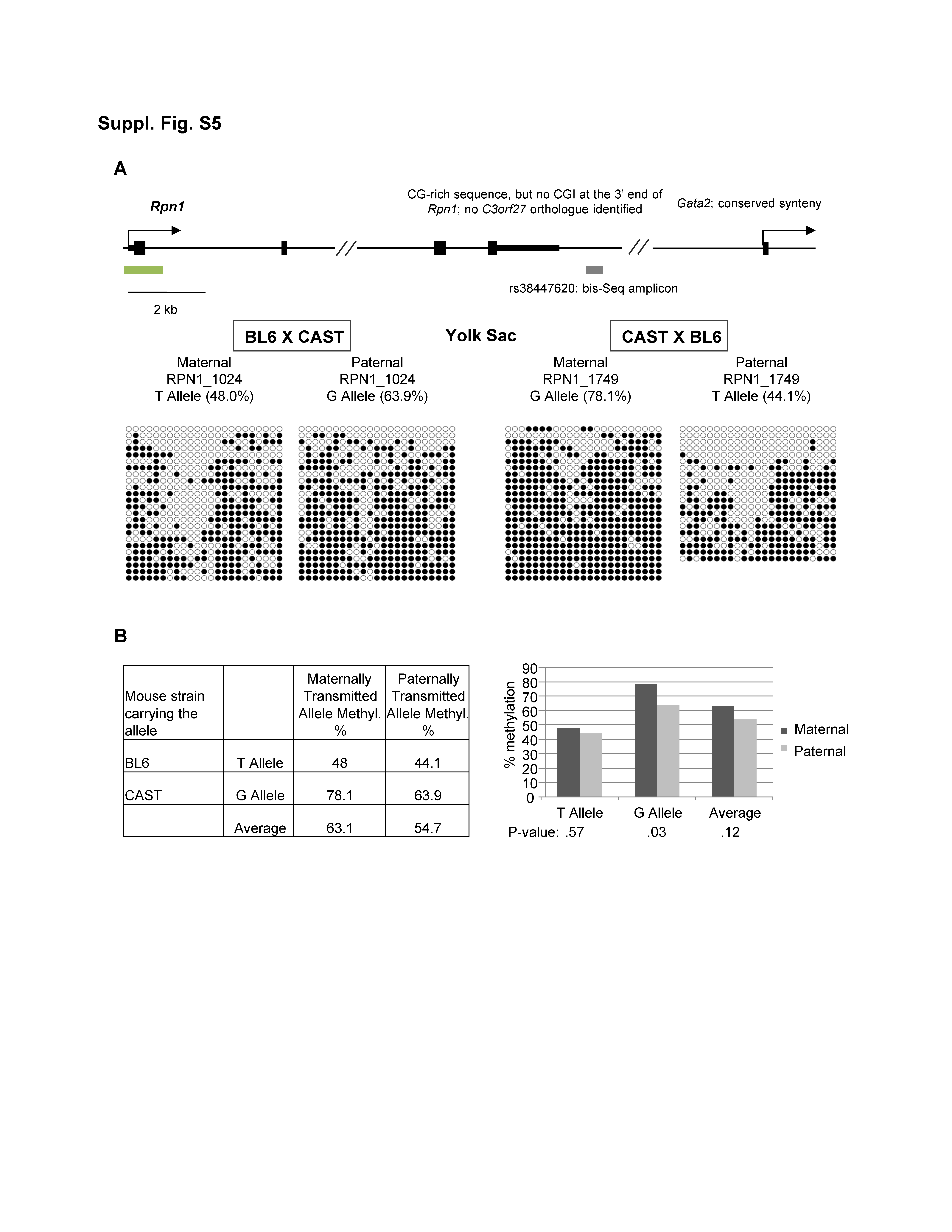

Supplement: Figure S5 — CpG methylation patterns in the mouse C3orf27-Rpn1 region are determined by a cis-acting haplotype effect superimposed on weak parental imprinting. A, Map of the region of the mouse genome that is orthologous to the human C3orf27-RPN1 region and bisulfite sequencing of the Rpn1 downstream region. There is only partial conservation of synteny. Primers used for bisulfite PCR were GGGTTAAGGGATTGTTTAAATAGTTA and ACCAAACCTTTAAACCAAAAAAAAC, which amplify a 390 bp CG-rich sequence that does not meet formal criteria for a CGI. B, Percent methylation of each allele as a function of strain-of-origin and parent-of-origin. T-tests were performed on the dataset consisting of the methylation values for each of the allele-specific bisulfite clones in each of the indicated yolk sac samples with known strain- and parent-of-origin. (TIFF) [file pgen.1003622.s005.tiff]

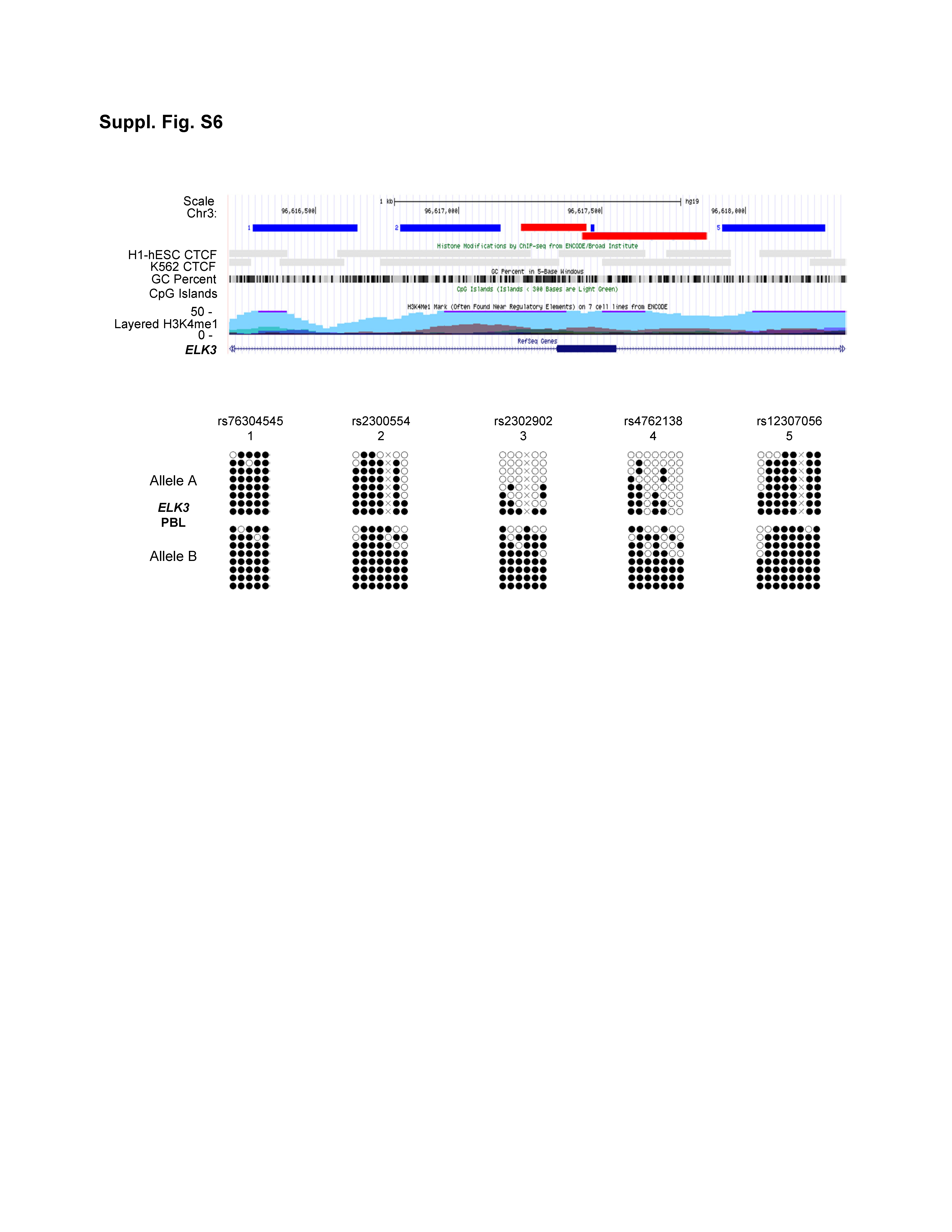

Supplement: Figure S6 — Local methylation mapping of the ELK3 DMR. Bisulfite sequencing of heterozygous PBL samples for multiple ELK3 amplicons shows the specific range of sequence dependent ASM in this locus, spanning 225 bp (chr12: 96,617,249–96,617,474). (TIFF) [file pgen.1003622.s006.tiff]

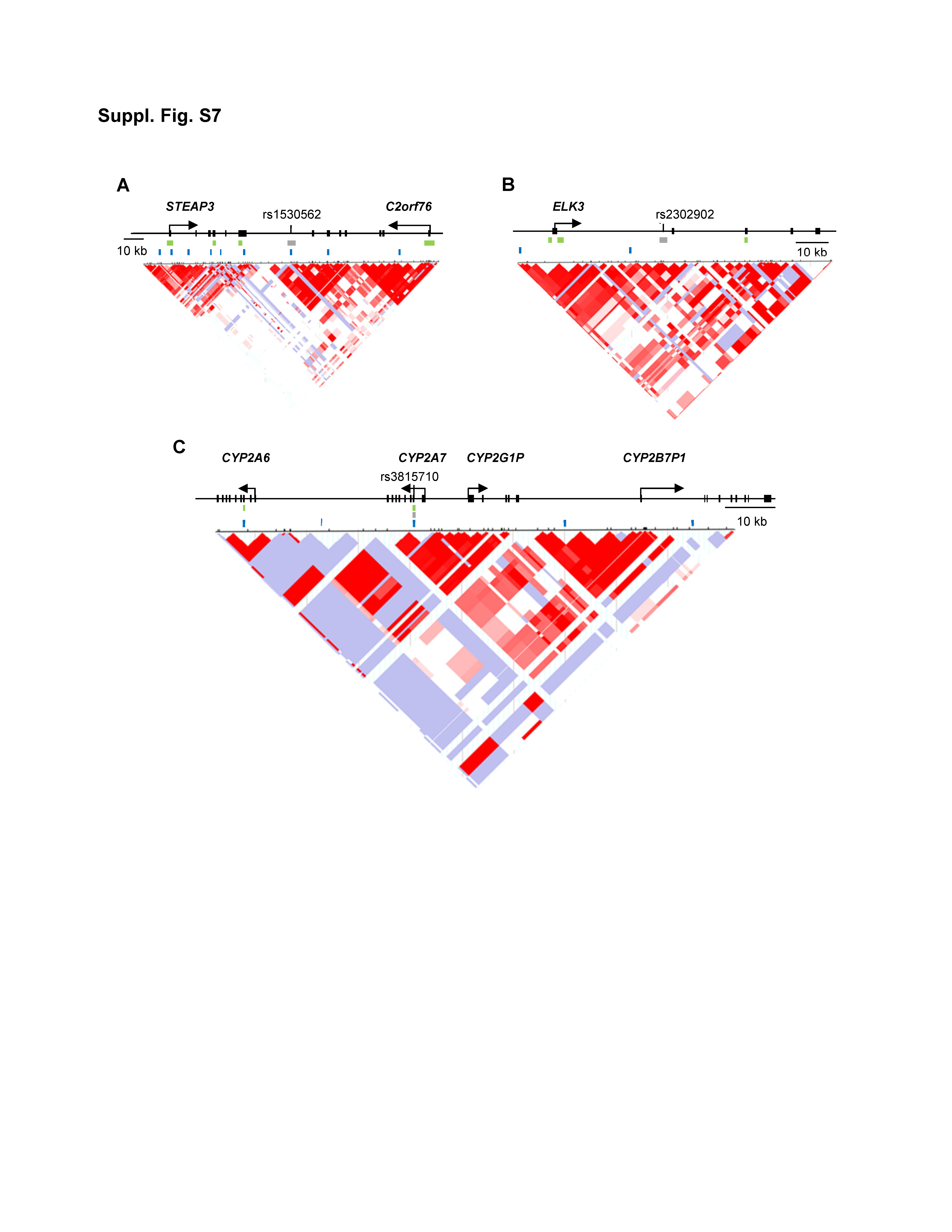

Supplement: Figure S7 — Haplotype blocks aligned to the loci with non-imprinted sequence dependent ASM using International HapMap Project (http://hapmap.ncbi.nlm.nih.gov/). In the gene maps the grey bars indicate the index DMRs, green bars indicate CGIs, blue bars are CTCF sites and the black rectangles are gene exons. Each of the DMRs is within a block of strong linkage disequilibrium. The STEAP3-C2orf76 DMR (A) and the CYP2A7 DMR (C) overlap CTCF binding sites, while the ELK3 DMR (B) , which has recurrent but less strong ASM, does not. (TIFF) [file pgen.1003622.s007.tiff]

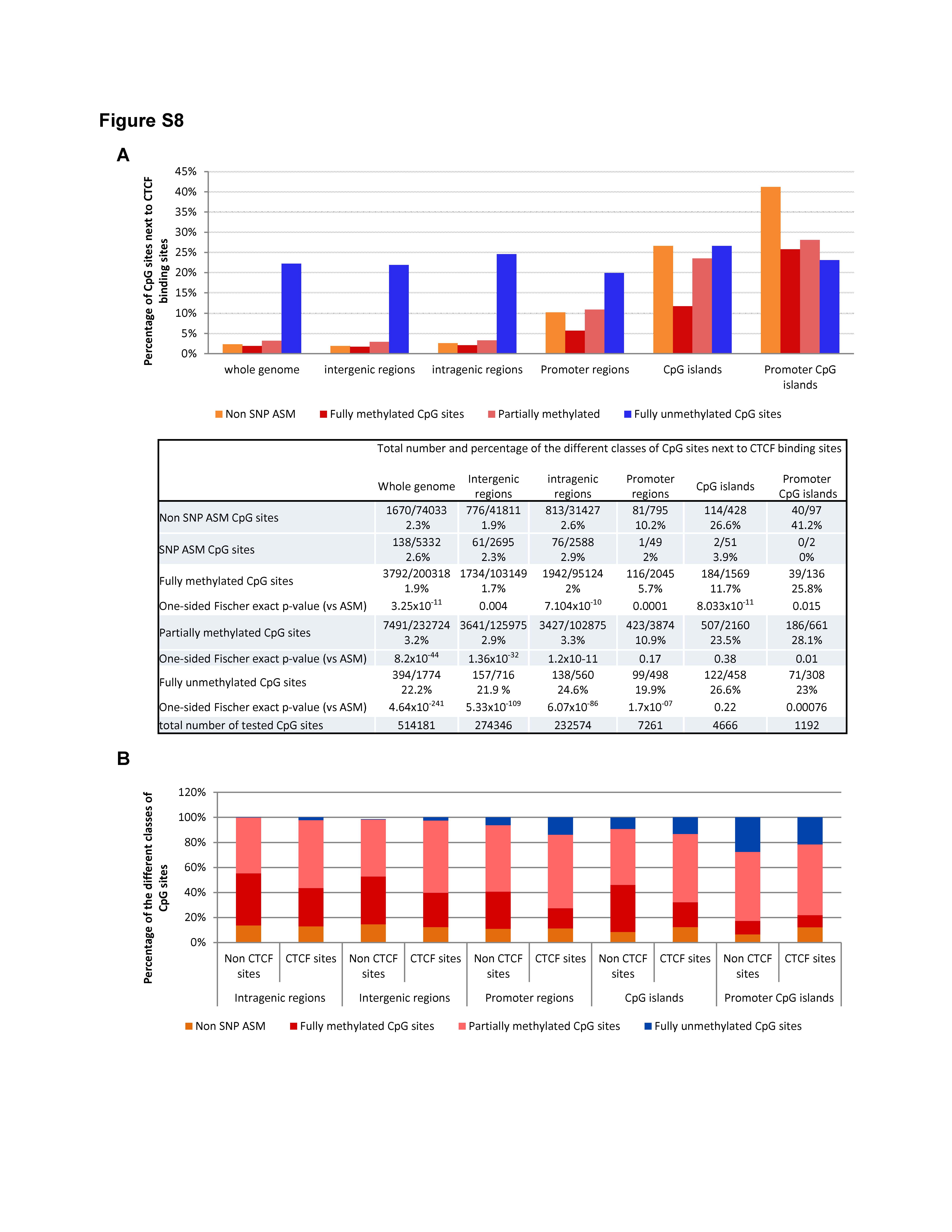

Supplement: Figure S8 — Association of ASM CpGs with particular locations of CTCF binding sites in H1-hESC. A, Bar graphs and table showing the percentage of the different classes of CpG sites in or near CTCF binding sites (in 500 bp windows centered on the CpG sites analyzed by Chen et al. [15]) in H1-ES cells. Classes of CpG sites (ASM as determined by Chen et al. [15], or fully, partially, or unmethylated, as determined by us) are color coded. Separate analyses have been performed for CpG sites in intergenic regions, intragenic regions, promoter regions, CGIs and promoter CGIs. The total numbers of sites and one sided Fischer exact p-values for the enrichment of CTCF sites, compared to CpGs that are fully methylated, partially methylated or fully unmethylated, are indicated in the table. B, Bar graphs showing the distribution of the different classes of CpG sites (ASM, fully methylated, partially methylated, fully unmethylated), with or without a CTCF binding site in a 500 bp window centered on the CpG. The same color codes as in panel A are used for ASM, fully methylated, partially methylated and fully unmethylated CpG sites. Separate analyses have been performed for CpG sites in intergenic regions, intragenic regions, promoter regions, CGIs and promoter CGIs. (TIFF) [file pgen.1003622.s008.tiff]

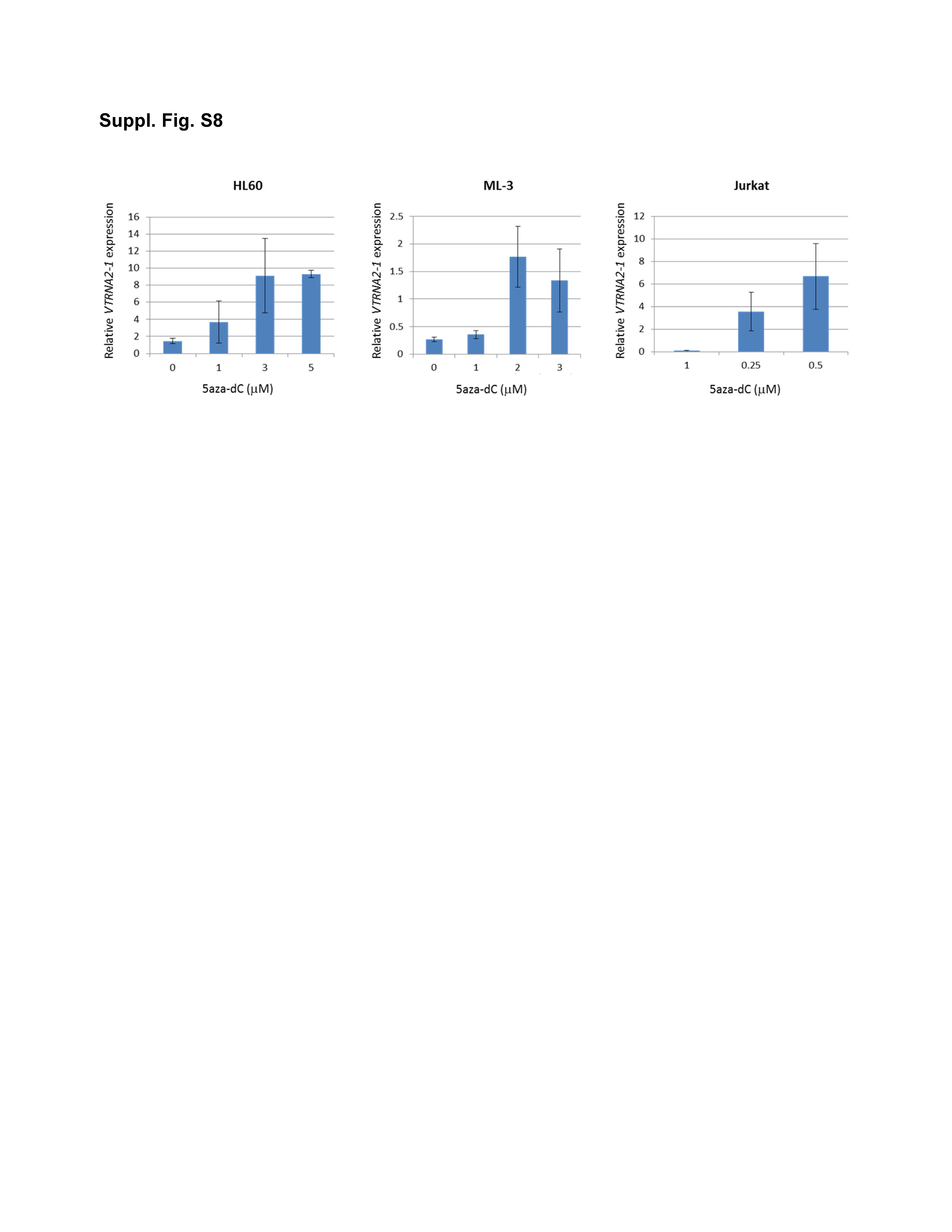

Supplement: Figure S9 — Effects of the demethylating drug 5aza-dC on expression of VTRNA2-1 RNA in HL60, ML3 and Jurkat cell lines. In each cell line, cells were treated with the indicated concentrations for three days. Increased expression of VTRNA2 is observed in all three cell lines and a reduction in methylation was confirmed in Jurkat cells for the index region DMR (data not shown). (TIFF) [file pgen.1003622.s009.tiff]
